# Overexpression of PRDM16 attenuates acute kidney injury progression: genetic and pharmacological approaches

**DOI:** 10.1002/mco2.737

**Published:** 2024-09-21

**Authors:** Xiaozhou Li, Fang Xu, Pan Zhang, Liufeng Mao, Yong Guo, Huiling Li, Yuxing Xie, Yijian Li, Yingjun Liao, Junxiang Chen, Donghai Wu, Dongshan Zhang

**Affiliations:** ^1^ Department of Emergency Medicine The Second Xiangya Hospital Central South University Changsha Hunan China; ^2^ Emergency Medicine and Difficult Diseases Institute,Department of Emergency Medicine The Second Xiangya Hospital Central South University Changsha Hunan China; ^3^ Department of Epidemiology and Health Statistics Xiangya School of Public Health Central South University Changsha Hunan China; ^4^ CAS Key Laboratory of Regenerative Biology, Joint School of Life Sciences Guangzhou Medical University Guangzhou China; ^5^ Department of Organ Procurement Organization The Second Xiangya Hospital Central South University Changsha Hunan China; ^6^ Department of Ophthalmology The Second Xiangya Hospital Central South University Changsha Hunan China; ^7^ Department of Urology The Second Xiangya Hospital Central South University Changsha Hunan China; ^8^ Department of Anesthesiology The Second Xiangya Hospital Central South University Changsha Hunan China; ^9^ Department of Nephrology The Second Xiangya Hospital Central South University Changsha Hunan China; ^10^ CAS Key Laboratory of Regenerative Biology, Joint School of Life Sciences,Guangzhou Institutes of Biomedicine and Health Chinese Academy of Sciences Guangzhou China

**Keywords:** AKI, apoptosis, PRDM16

## Abstract

Acute kidney injury (AKI) presents as a condition marked by a sudden and rapid decrease in kidney function over a short timeframe, resulting from diverse causes. As a transcription factor, PR domain‐containing 16 (PRDM16), has recently been implicated in brown fat biogenesis and heart diseases. Our recent works indicated that PRDM16 could suppress the occurrence of renal interstitial fibrosis in diabetic kidney disorder. Nonetheless, the effect and regulatory mechanism of PRDM16 in AKI remain elusive. Our study demonstrated that PRDM16 inhibited apoptosis induced by ischemic/reperfusion (I/R) in BUMPT (Boston University mouse kidney proximal tubular) cells and HK‐2(Human Kidney‐2) cells. Mechanistically, PRDM16 not only bound to the promoter region of S100 Calcium Binding Protein A6 (S100A6)and upregulated its expression but also interacted with its amino acids 945–949, 957–960, and 981–984 to suppress the p38MAPK and JNK axes via inhibition of PKC‐η activity and mitochondrial reactive oxygen species (ROS) production. Furthermore, cisplatin‐ and I/R‐stimulated AKI progression were ameliorated in PRDM16 proximal‐tubule‐specific knockin mice, whereas exacerbated in PRDM16 knockout proximal‐tubule‐specific mice). Moreover, we observed that formononetin ameliorated I/R‐ and cisplatin‐triggered AKI progression in mice. Taken together, these findings reveal a novel self‐protective mechanism in AKI, whereby PRDM16 regulates the S100A6/PKC‐η/ROS/p38MAPK and JNK pathways to inhibit AKI progression.

## Graphical abstract

We observed that PRDM16 ameliorated I/R and cisplatin‐induced progression of mice AKI by regulating the PRDM16/S100A6/PKC‐η/ROS/p38MAPK and JNK axes. These data indicate that a new self‐protection mechanism of AKI is the inhibition of its progression by PRDM16, which regulates the S100A6/PKC‐η/ROS/p38MAPK and JNK pathways, and furthermore, PRDM16 represents a potential therapeutic target for AKI.

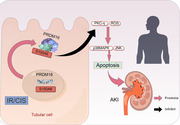

## 1 INTRODUCTION

Acute kidney injury (AKI) manifests as a rapid elevation in serum creatinine (Cr) levels, diminished urine output, or both, affecting approximately 10−15% of hospitalized individuals and over 50% of critically ill patients.^1^ AKI is usually caused by multiple factors, including ischemia, drug toxicity, and sepsis.^2^ Among its key pathological features are renal tubules exhibiting either lethal or sublethal injury.^3^ Over the past three decades, tubular cell apoptosis has emerged as a primary contributor to tubular cell death.^4^, ^5^, ^6^ Recent investigations have highlighted the significance of self‐protection pathways as therapeutic targets for diverse conditions, including tumor, brain ischemia, and nephrotoxicity.^7^, ^8^, ^9^, ^10^, ^11^ Some studies have demonstrated that enhancing HIF‐1α and superoxide can mitigate the progression of ischemic AKI, respectively.^12^, ^13^ Hence, there is a pressing need to explore the self‐protection mechanisms of tubular cell apoptosis, identify novel prevention and treatment targets, and develop effective drugs for treating AKI.

PRDM16 (PRD1‐BF1‐RIZ1‐homologous‐domain‐containing‐16) is a transcriptional factor primarily associated with the regulation and functionality of beige and brown fat, as well as cardiac development and hematopoiesis.^14^, ^15^, ^16^, ^17^, ^18^, ^19^, ^20^, ^21^ Specifically, PRDM16 influences cardiac development through Hand1 and Tbx5 and then regulates myocardial structures.^22^ In addition, PRDM16 plays a role in hematopoietic stress response and thymic degeneration.^23^ Several studies have indicated that PRDM16 suppresses the progression of kidney tumors and lung adenocarcinomas.^15^, ^24^ Interestingly, one study reported that PRDM16 was expressed during kidney development.^25^ Our recent study verified the antifibrotic role of PRDM16 in diabetic kidney disease.^26^ Nonetheless, the effect and regulatory mechanisms of PRDM16 in AKI are still unclarified.

In this research, we present the first evidence of PRDM16 protective role against apoptosis in BUMPT cells during injury induced by ischemia/reperfusion (I/R). Mechanistically, PRDM16 directly upregulated and interacted with S100 Calcium Binding Protein A6 (S100A6), subsequently inhibiting the activation of the PKC‐η/ROS/p38MAPK and JNK axes. Furthermore, I/R‐ and cisplatin‐stimulated AKI in mice were exacerbated in proximal‐tubule‐specific PRDM16 knockout (PRDM16‐PT‐KO) mice, while attenuated in proximal‐tubule‐specific PRDM16 knockin (PRDM16‐PT‐KI) mice. Additionally, we demonstrated that formononetin ameliorated I/R‐ and cisplatin‐stimulated AKI in mice.

Our findings imply that PRDM16 is a new attractive therapeutic target for I/R‐ and cisplatin‐stimulated AKI. Moreover, formononetin acts on PRDM16 and then prevents the progression of I/R‐ and cisplatin‐stimulated AKI.

## 2 RESULTS

### 2.1 PRDM16 is triggered by I/R in BUMPT cells and mouse kidney and protects against I/R‐triggered necrosis and apoptosis in BUMPT cell line

**FIGURE 1:**
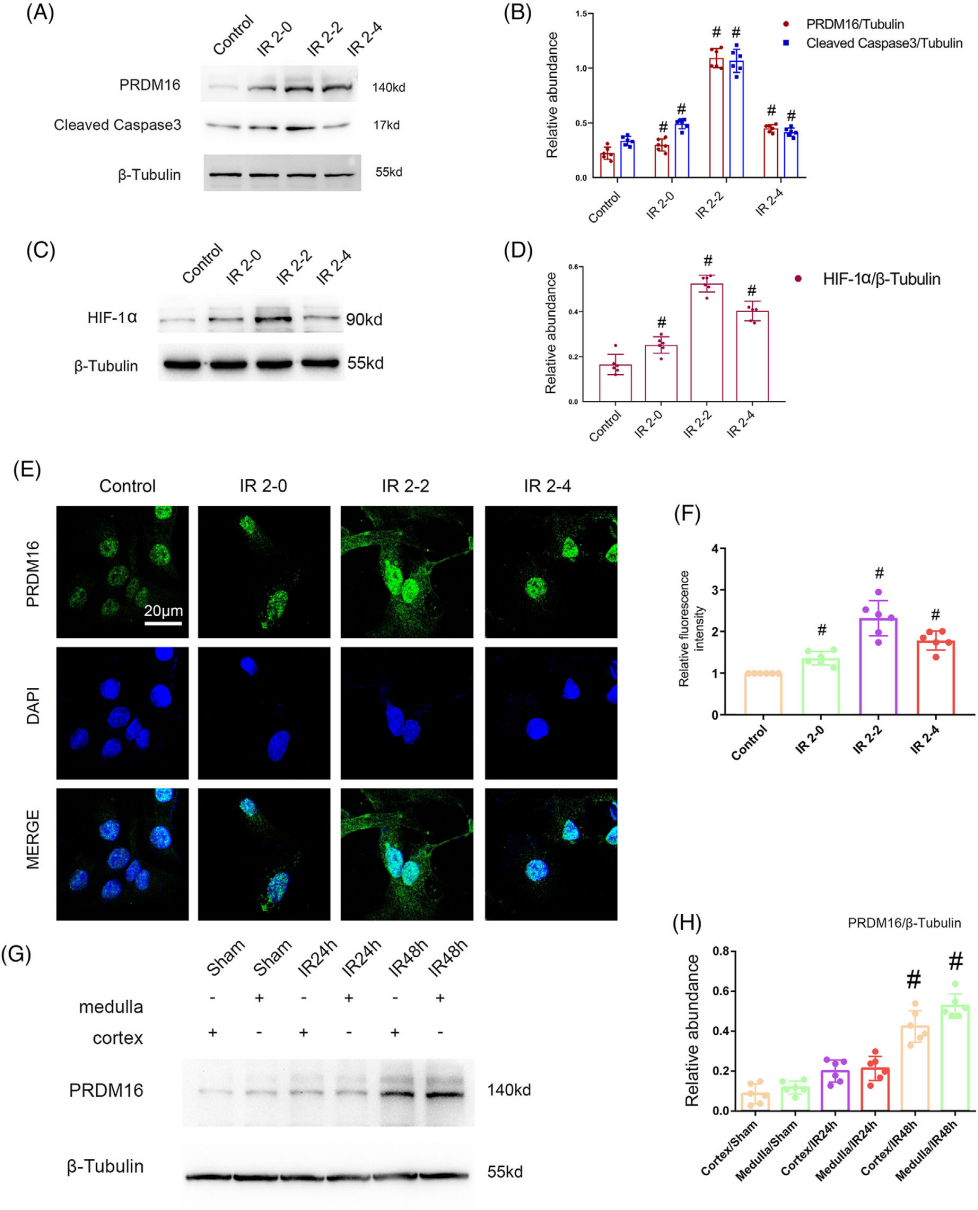
PRDM16 is triggered by I/R in BUMPT cellsand mouse kidney. BUMPT cells was treated with 1.5 µM calcium and 10 µM antimycin A for 2 h and recovered for 0, 2, and 4 h. The I/R (ischemic/reperfusion) mouse model was stimulated by ischemia for 28 min and reperfusion for 24 and 48 h. (A) The immunoblot analysis of PRDM16, cleaved‐caspase3, and β‐tubulin in BUMPT cellssubjected to the I/R model. (B) Quantification of protein blots. (C and D) BUMPT cells was treated with 1.5 µM calcium and 10 µM antimycin for 2 h and recovered for 0, 2, and 4 h. (C) Immunoblotting of HIF1a, and β‐tubulin. (D) Quantification of protein blots. (E) Immunofluorescence analysis of the expression/localization of PRDM16 in BUMPT cells. (F) Relative fluorescence intensity. (G) Immunoblotting of PRDM16 and β‐tubulin in C57BL/6 mice in the I/R model. (H) Quantification of protein blots. Original magnification ×600. Scale bar: 20 µM. Means ± SD (*n* = 6). #*p* < 0.05 versus controls.

We initially investigated whether PRDM16 was stimulated by I/R. First, BUMPT cells underwent treatment with antimycin and a calcium ionophore (i.e., ischemic injury) simulating ischemic injury, for 2 h, and then reperfusion for 0−4 h. The immunoblotting findings demonstrated a progressive upregulation of PRDM16 and cleaved‐caspase3 expression, peaking at 2 h postreperfusion, and then declining by 4 h postreperfusion (Figure 1A,B). The expression trend of HIF‐1α is similar with that of PRDM16 (Figure 1C,D). These findings were corroborated by immunofluorescence staining of PRDM16, which showed a similar temporal pattern (Figure 1E,F). Furthermore, the immunofluorescence results indicated predominant nuclear localization of PRDM16 in BUMPT cells, with only a small amount observed in the cytoplasm. Subsequently, C57BL/6 mice underwent I/R (28 min ischemia/24–48 h reperfusion). Immunoblot analysis revealed a notable increase in PRDM16 expression at 24 h postreperfusion, peaking at 48 h postreperfusion. Additionally, PRDM16 expression in the kidney cortex was weaker compared with the medulla (Figure 1G,H). Altogether, these data demonstrate that PRDM16 is induced in the ischemic AKI model in both BUMPT cellsand mice.

**FIGURE 2:**
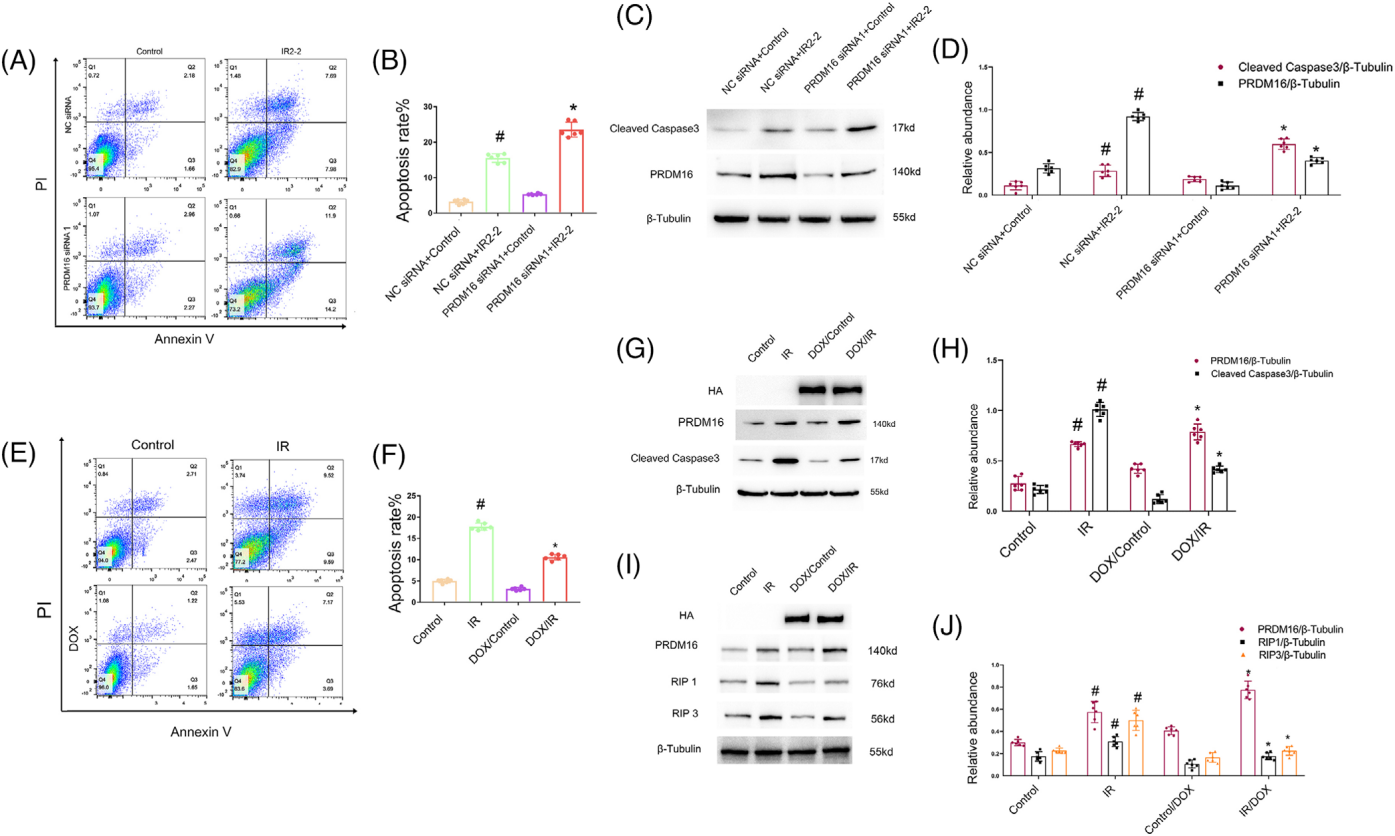
PRDM16 negatively regulates the I/R‐stimulated BUMPT cells apoptosis. PRDM16 siRNA was transfected into BUMPT cells, which was subsequently exposed to ischemia for 2 h and recovery for 2 h. In addition, the cell line with stable PRDM16‐RFP expression was treated with ATP depletion for 2 h and recovery for 2 h with or without DOX (doxycycline) for 48 h. (A) FCM (flow cytometry) analysis. (B) Quantitative data for apoptosis. (C) Immunoblotting of PRDM16, cleaved‐caspase3, and β‐tubulin. (D) Quantification of protein bands. (E) Flow cytometry analysis. (F) Quantitative data for apoptosis. (G) Immunoblotting of HA, PRDM16, cleaved‐caspase3, and β‐tubulin. (H) Quantification of protein bands. (I and J) The cell line with stable PRDM16‐RFP expression was treated with ATP depletion for 2 h and recovery for 2 h with/without doxycycline (DOX) for 48 h. (I) Immunoblotting of HA, PRDM16, RIP1, RIP3, and β‐tubulin. (J) Quantification of protein bands. Means ± SD (*n* = 6). #*p* < 0.05 versus NC siRNA plus controls. *p < 0.05 versus NC siRNA plus IR 2‐2.

We further investigated the roles of PRDM16 in I/R‐triggered BUMPT cells death. First, BUMPT cells were transfected with PRDM16 siRNA1 or 2 or scramble siRNA, followed by I/R (2 h ischemia/2 h reperfusion). Flow cytometry (FCM) analysis indicated that PRDM16 siRNA1 notably increased I/R‐stimulated BUMPT apoptosis (Figure 2A,B). Immunoblotting analysis indicated that PRDM16 expression was obviously suppressed, whereas that of cleaved‐caspase3 was noticeably increased by PRDM16 siRNA1 under both basal and ischemic injury conditions (Figure 2C,D). Similarly, immunoblotting analysis demonstrated that PRDM16 siRNA2 remarkably suppressed PRDM16 expression while markedly increasing cleaved caspase3 expression under basal and ischemic injury conditions (Figure S20A,B). Subsequently, we established a doxycycline (DOX)‐mediated HA‐PRDM16 stable cell line and exposed the cells to I/R (2 h ischemia/2 h reperfusion). FCM analysis revealed that DOX‐mediated HA‐PRDM16 overexpression markedly attenuated I/R‐stimulated BUMPT cells apoptosis (Figure 2E,F). Immunoblotting analysis indicated that DOX markedly induced HA expression, while significantly suppressing cleaved‐caspase3 expression under both basal and ischemic injury conditions (Figure 2G,H). In addition, DOX‐mediated HA‐PRDM16 overexpression noticeably ameliorated the expression of necrosis‐associated genes RIP1 and RIP3 in I/R‐induced BUMPT cell line (Figure 2I,J). Multiple studies have shown that RIP1 and RIP3 are associated with programmed necrosis.^27^, ^28^ These findings collectively demonstrate that PRDM16 acts as a protective factor against apoptosis and necrosis in BUMPT cells following I/R injury.

### 2.2 PRDM16 positively regulates and interacts with S100A6 in BUMPT cell line and mouse kidney under basal and I/R conditions

**FIGURE 3:**
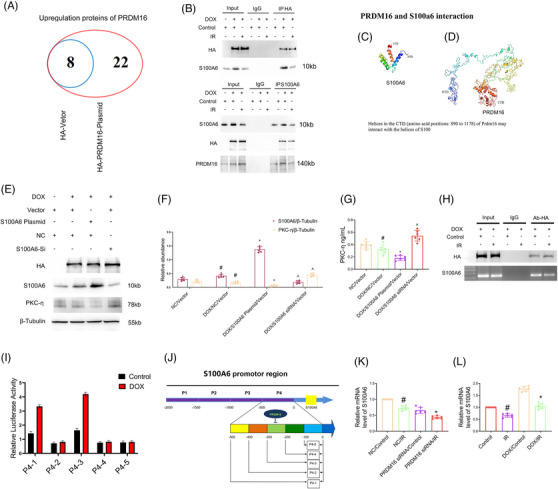
PRDM16 positively regulates and interacts with S100A6 in BUMPT cell line under baseline and I/R conditions. (A) Changes in the number of upregulated genes between (more than twofold changes) HA‐vector versus HA‐PRDM16 plasmids. (B) The cell lysate was harvested for reciprocal coimmunoprecipitation of HA and S100A6 (S100 Calcium Binding Protein A6) in the cell line with stable PRDM16‐RFP expression exposed to ATP depletion. (C and D) The prediction model of the PRDM16 and S100A6 interaction. (E) Immunoblot analysis of HA, S100A6, PKC‐η, and β‐tubulin. (F) Quantification of protein bands. (G) Concentration of PKC‐η by ELISA. (H) ChIP assays showed that PRDM16 bound to S100A6 promoter region. (I) Luciferase reporter assay demonstrated that PRDM16 could bind to a specific S100A6 promoter region in the cell line with stable PRDM16‐RFP expression. (J) Mapping of PRDM16 binding sites in S100A6 promoter region. (K) RT‐qPCR of S100A6 mRNA. (L) RT‐qPCR of S100A6 mRNA. Means ± SD (*n* = 6). #*p* < 0.05 versus DOX plus NC. *p < 0.05 versus DOX S100A6 plasmid plus DOX. ^*p* < 0.05 versus DOX S100A6 siRNA plus DOX S100A6 plasmid.

To further investigate the protection mechanism of PRDM16 for BUMPT cells apoptosis caused by ischemic injury, BUMPT cell line were transfected with HA empty plasmid and HA‐PRDM16 plasmid and cultured for 24 h. The cell lysate was harvested and underwent immunoprecipitation (IP) using HA antibody. The IP products were then collected for mass spectrometer (MS) evaluation. Subsequently, we identified 22 proteins that may interact with PRDM16 (Figure 3A and Table S1). Among them, S100A6 was previously shown to have the function of antagonizing cardiomyocyte apoptosis induced by hypoxia reoxygenation.^29^ Based on this, we hypothesize that PRDM16 interacts with S100A6 to mitigate the progression of AKI. To investigate whether PRDM16 indeed interacts with S100A6, we conducted IP experiments. The results showed that the anti‐HA antibody precipitated HA, PRDM16, and S100A6 proteins in the whole lysates of the DOX and I/R plus DOX groups, but not in the control group. In addition, anti‐S100A6 precipitated both S100A6 and PRDM16 in the whole lysates of the control, DOX, and I/R plus DOX groups of BUMPT cell line, but only HA in the DOX and I/R plus DOX groups (Figure 3B). The structure of S100A6 containing the amino‐terminal domain and carboxyl‐terminal domain (CTD) is shown in Figure 3D. Furthermore, soft prediction indicated that the CTD (amino acids 890−1178) of PRDM16 might interact with S100A6 (Figure 3C). To confirm this prediction, we constructed 3 plasmids for HA‐PRDM16 with amino acids 1−889, 890−1178, and 1−1178, which were then transfected into BUMPT cell line, followed by treatment with/without I/R (2 h/2 h). The anti‐HA antibody could precipitate S100A6 in the plasmid containing PRDM16 with amino acids 890−1178 and 1−1178, but not containing with amino acids 1−889. This result shows that S100A6 interacts with PRDM16 region that contains amino acids 890−1178 (Figure S21A). To identify the PRDM16 region interacting with S100A6, we used the HA‐Tag of PRDM16 plasmids with Δ945–949, 957−960, 981−984, Δ1089–1092, 1103−1107, Δ945–949, 957−960, 981−984, 1089−1092, and 1103−1107. The IP findings indicated that the anti‐HA antibody could precipitate S100A6 only with the Δ1089–1092 and 1103−1107 plasmids, but not with the Δ945–949, 957−960, 981−984 and Δ945–949, 957−960, 981−984, 1089−1092, and 1103−1107 plasmids (Figure S21B). This further confirms that S100A6 interacts with amino acids 945−949, 957−960, and 981−984 of PRDM16. In summary, these findings imply that PRDM16 directly regulates the expression of S100A6 and that amino acids 945−949, 957−960, and 981−984 of PRDM16 are involved in the interaction with S100A6.

Herein, we also found that PKC‐η expression was suppressed in HA‐PRDM16 with DOX BUMPT cell line when S100A6 was overexpressed. In contrast, PKC‐η expression was increased in HA‐PRDM16 with DOX BUMPT cell line when S100A6 was knocked down (Figure 3E,F). This observation was further confirmed by the ELISA detection of PKC‐η activity (Figure 3G), suggesting that the interaction of PRDM16 and S100A6 might affect the PKC‐η activity. Next, we explored whether PRDM16 could regulate the expression of S100A6. The stable HA‐PRDM16‐RFP‐expressing cells were exposed to control or I/R with DOX, and then underwent ChIP assays using an anti‐HA antibody. The ChIP results indicated that PRDM16 interacted with binding site 4 (−500–0 bp) in the S100A6 promoter (Figure 3H). Following this, we constructed five segments of S100A6‐promoter‐luciferase‐reporter plasmids (P4 1−5) and conducted a luciferase reporter assay to identify the linking region between PRDM16 and S100A6. When the cell line with consistent HA‐PRDM16‐RFP expression underwent transfection with S100A6‐promoter plasmids (P4 1−5) and treated with/without DOX for 24 h, endogenous PRDM16 did not notably increase the S100A6 promoter activities of P4 1 and 3 or P4 2‐4‐5 in any of the DOX treatment groups. However, after DOX treatment, the S100A6 promoter activities of P4 1 & 3, but not P4 2, 4, 5, were further increased by PRDM16 compared with the control groups. Thus, the luciferase reporter assays indicated that PRDM16 interacted with (−300−200 bp) in the S100A6 promoter (Figure 3I). An abridged diagram (Figure 3J) illustrated that PRDM16 could directly bind to the S100A6 promoter. Interestingly, PRDM16 knockdown inhibited the mRNA level of S100A6 during I/R treatment, while PRDM16 overexpression upregulated that of S100A6 (Figure 3K,L). In addition, our study demonstrated that Pifithrin‐a, a commonly used p53 inhibitor, attenuated the I/R‐triggered suppression of the mRNA and protein level of S100A6 in BUMPT and HK‐2 cells (Figure S19A–C). This finding further supports the notion that S100A6 is positively regulated by PRDM16 and negatively regulated by p53.

A colocalization analysis was performed to verify the results of the IP experiments. Immunofluorescence and confocal microscopy of PRDM16 and S100A6 revealed their colocalization in the cytoplasms of the BUMPT and kidney tubular cell lines of sham mouse kidney. After ischemic treatment, the PRDM16 signal increased, whereas the S100A6 signal decreased. However, the colocalization signal was increased mainly in the cytoplasm and partially in the nucleus of BUMPT cells following I/R injury, and in the kidney tubular cells of mouse kidney post I/R injury (Figure S22A,B). Collectively, the findings further support that PRDM16 interacts with S100A6 predominantly in the cytoplasm and to some extent in the nucleus.

### 2.3 S100A6 attenuates apoptosis in BUMPT cells mediated by I/R injury via inactivation of the PKC‐η/p38MAPK and JNK signaling pathways and mediates the antiapoptotic properties of PRDM16

**FIGURE 4:**
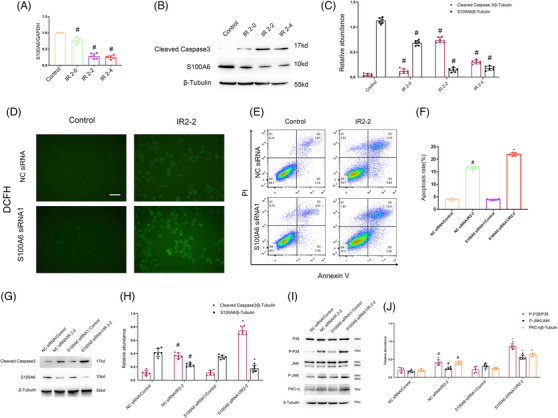
Knockdown of S100A6 aggravates the I/R‐stimulated expression levels of apoptotic genes via activation of PKC‐η/ROS/p38 MAPK and JNK in BUMPT cells. The S100A6 (S100 Calcium Binding Protein A6) siRNA was transfected into BUMPT cells, which was subsequently exposed to ischemia for 2 h and recovered for 2 h. (A) RT‒qPCR of S100A6. (B) Immunoblotting of S100A6, cleaved‐caspase3, and β‐tubulin. (C) Quantification of protein bands. (D) ROS (reactive oxygen species) level assessed by DCFH (dichlorodihydrofluorescein). (E) FCM (flow cytometry) analysis. (F) Quantitative data for apoptosis. (G) Immunoblotting of cleaved‐caspase3, S100A6, and β‐tubulin. (H) Quantification of protein bands. (I) Immunoblotting of p‐P38MAPK, P38MAPK, p‐JNK, JNK, PKC‐η, and β‐tubulin. (J) Quantification of protein bands. Magnification ×400. Scale bar: 100 µM. Means ± SD (*n* = 6). #*p* < 0.05 versus scramble plus controls. *p < 0.05 versus scramble plus IR.

**FIGURE 5:**
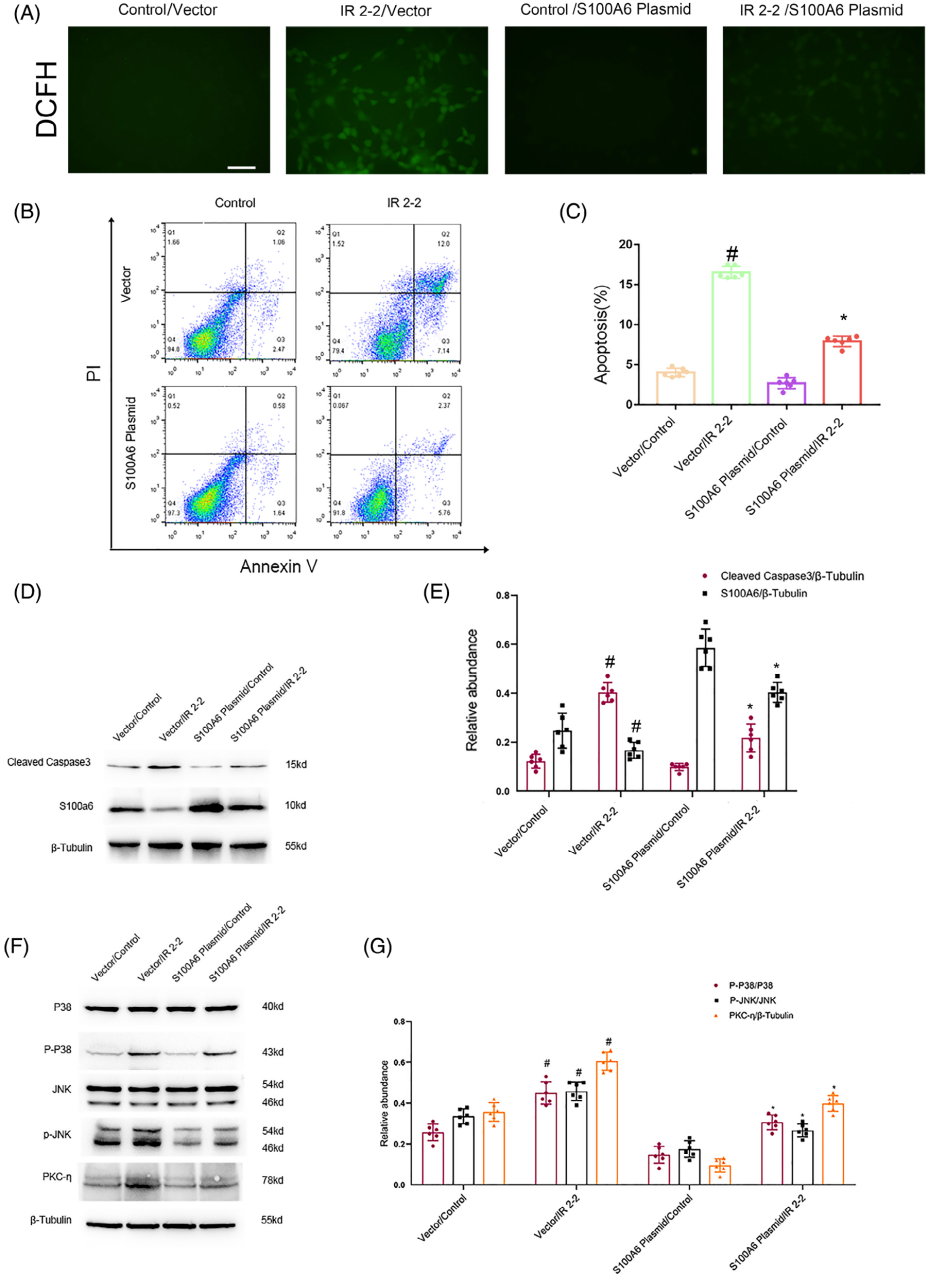
Overexpression of S100A6 attenuates the I/R‐stimulated expression levels of apoptotic genes via inactivation of PKC‐η/ROS/p38MAPK and JNK in BUMPT cells. The S100A6 (S100 Calcium Binding Protein A6) plasmid was transfected into BUMPT cells, which was subsequently exposed to ischemia for 2 h and recovered for 2 h. (A) ROS (reactive oxygen species) level assessed by DCFH (dichlorodihydrofluorescein). (B) FCM (flow cytometry) analysis. (C) Quantitative data for apoptosis. (D) Immunoblotting of cleaved‐caspase3, PRDM16, S100A6, and β‐tubulin. (E) Quantification of protein bands. (F) Immunoblotting of P‐P38MAPK, P38MAPK, P‐JNK, JNK, PKC‐η, and β‐tubulin. (G) Quantification of protein bands. Magnification ×400. Scale bar: 100 µM. Means ± SD (*n* = 6). #*p* < 0.05 versus scramble plus controls. *p < 0.05 versus scramble plus IR.

We then explored the role and regulatory mechanism of S100A6 in the progression of I/R‐stimulated BUMPT cells death. The results from quantitative RT‐qPCR and immunoblotting demonstrated that the protein/mRNA levels of S100A6 were reduced by I/R 2/0 and attained the lowest point at I/R 2/2&4. This trend is opposite to the observed upregulation of cleaved‐caspase3 (Figure 4A–C). Additionally, DCFH assay revealed that S100A6 siRNA noticeably increased ROS production induced by IR, while S100A6 overexpression had the opposite effect (Figures 4D and 5A). FCM analysis showed that S100A6 siRNA1 markedly aggravated I/R‐stimulated BUMPT cells death (Figure 4E,F). Immunoblotting results indicated that both siRNA1 and siRNA2 of S100A6 markedly enhanced the activation of cleaved‐caspase3, p38MAPK, and JNK, as well as PKC‐η expression under basal and I/R conditions (Figures 4G,J and S20C–F). Conversely, these changes were mitigated by overexpression of the S100A6 plasmid (Figure 5B–G). In summary, the data suggest that S100A6 ameliorates I/R‐stimulated BUMPT cells death via inhibiting the PKC‐η/ROS/p38MAPK and JNK signaling pathways.

To validate whether the antiapoptotic function of PRDM16 in BUMPT cells was dependent on S100A6 during I/R‐induced injury, we conducted two experiments. First, analysis using DCFH and FCM revealed that knockdown of PRDM16 markedly exacerbated ROS production and apoptosis triggered by I/R in the BUMPT cells. Nevertheless, this effect was reversed upon S100A6 overexpression (Figure S3A–C). Additionally, immunoblotting results indicated that knockdown of PRDM16 significantly intensified the I/R‐stimulated upregulation of cleaved‐caspase3, p‐p38MAPK, p‐JNK, and PKC‐η expression by downregulating S100A6. Nevertheless, this effect was counteracted by overexpression of S100A6 (Figure S3D–G). Second, DCFH and FCM analysis showed that knockdown of S100A6 noticeably improved I/R‐stimulated ROS production and apoptosis in the BUMPT cell line without DOX treatment. Interestingly, this effect persisted under I/R conditions with DOX treatment (Figure S4A–C). Furthermore, the immunoblotting results demonstrated that knockdown of S100A6 significantly enhanced the increase in cleaved‐caspase3, p‐p38MAPK, and p‐JNK, as well as the expression of PKC‐η stimulated by I/R (Figure S4D–G). However, this effect was not alleviated by I/R treatment plus DOX conditions (Figure S4D–G). In summary, these observations imply that S100A6 plays a vital role in mediating the protective function of PRDM16 in BUMPT cells during I/R injury.

### 2.4 I/R‐ and cisplatin‐triggered AKI is aggravated in PRDM16‐PT‐KO mice while attenuated by PRDM16‐PT‐KI mice

**FIGURE 6:**
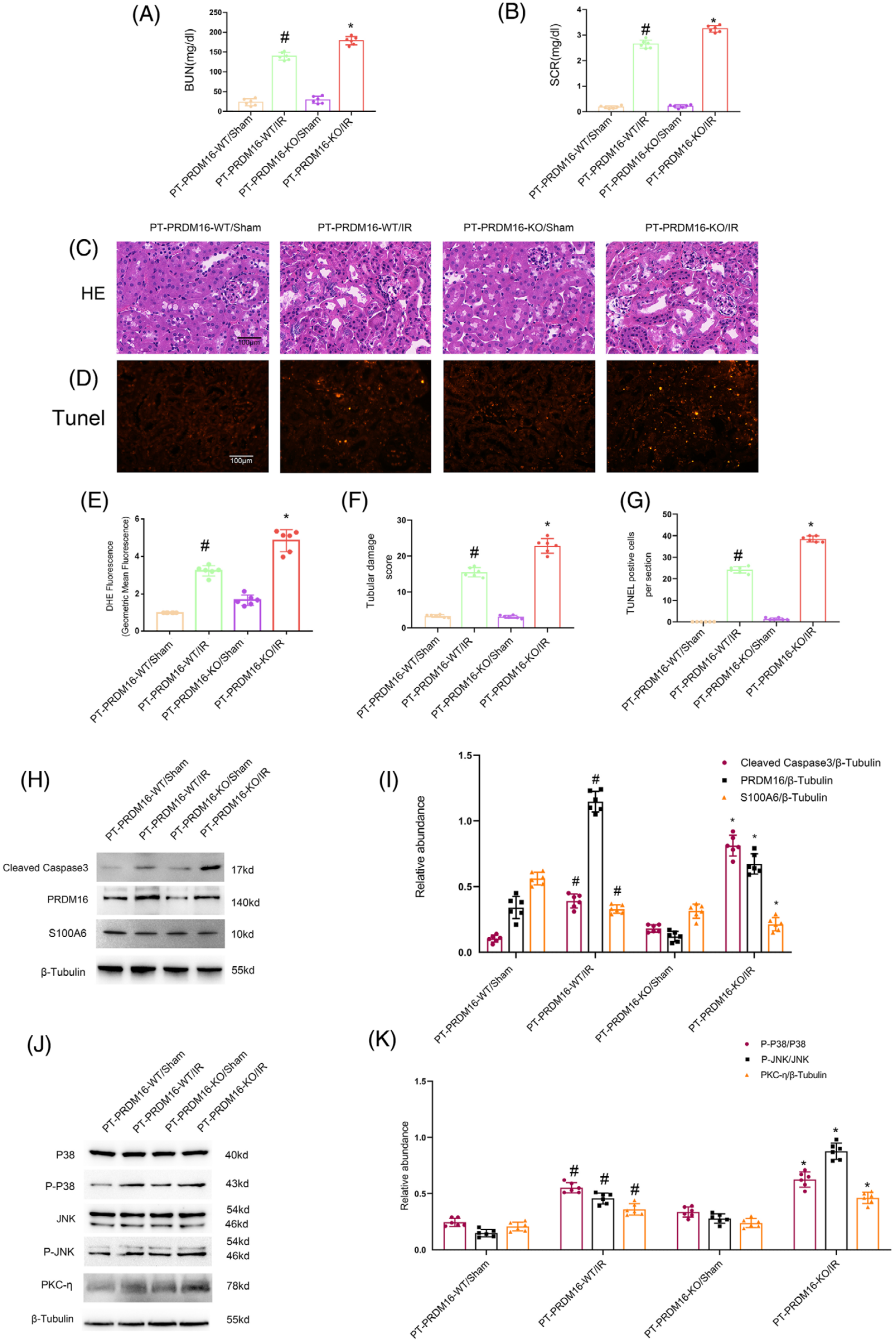
PRDM16‐PT‐KO aggravates the IR‐stimulated renal injury, tubular cell apoptosis via regulation S100A6/PKC‐η/ROS/p38MAPK and JNK axis. The bilateral kidney arteries of PRDM16‐PT‐KO (proximal tubule‐specific PRDM16 knockout mouse) and PRDM16‐PT‐WT mice were clamped for 28 min and subsequently released for 48 h to construct an I/R (ischemic/reperfusion) model. (A) BUN (blood urea nitrogen). (B) Serum creatinine. (C) H&E staining. (D) Representative regions with TUNEL‐positive staining. (E) Quantitative data for DHE (dihydroethidium). (F) Tubular damage scores. (G) Number of cells showing TUNEL positivity. (H) Immunoblotting of cleaved‐caspase3, PRDM16, S100A6, and β‐tubulin. (I) Quantification of protein bands. (J) Immunoblotting of p‐P38MAPK, P38MAPK, p‐JNK, JNK, PKC‐η, and β‐tubulin. (K) Quantification of protein bands. Magnification ×400. Scale bar: 100 µM. Means ± SD (*n* = 6). #*p* < 0.05 versus sham. *p < 0.05 versus PRDM16‐PT‐WT plus IR.

**FIGURE 7:**
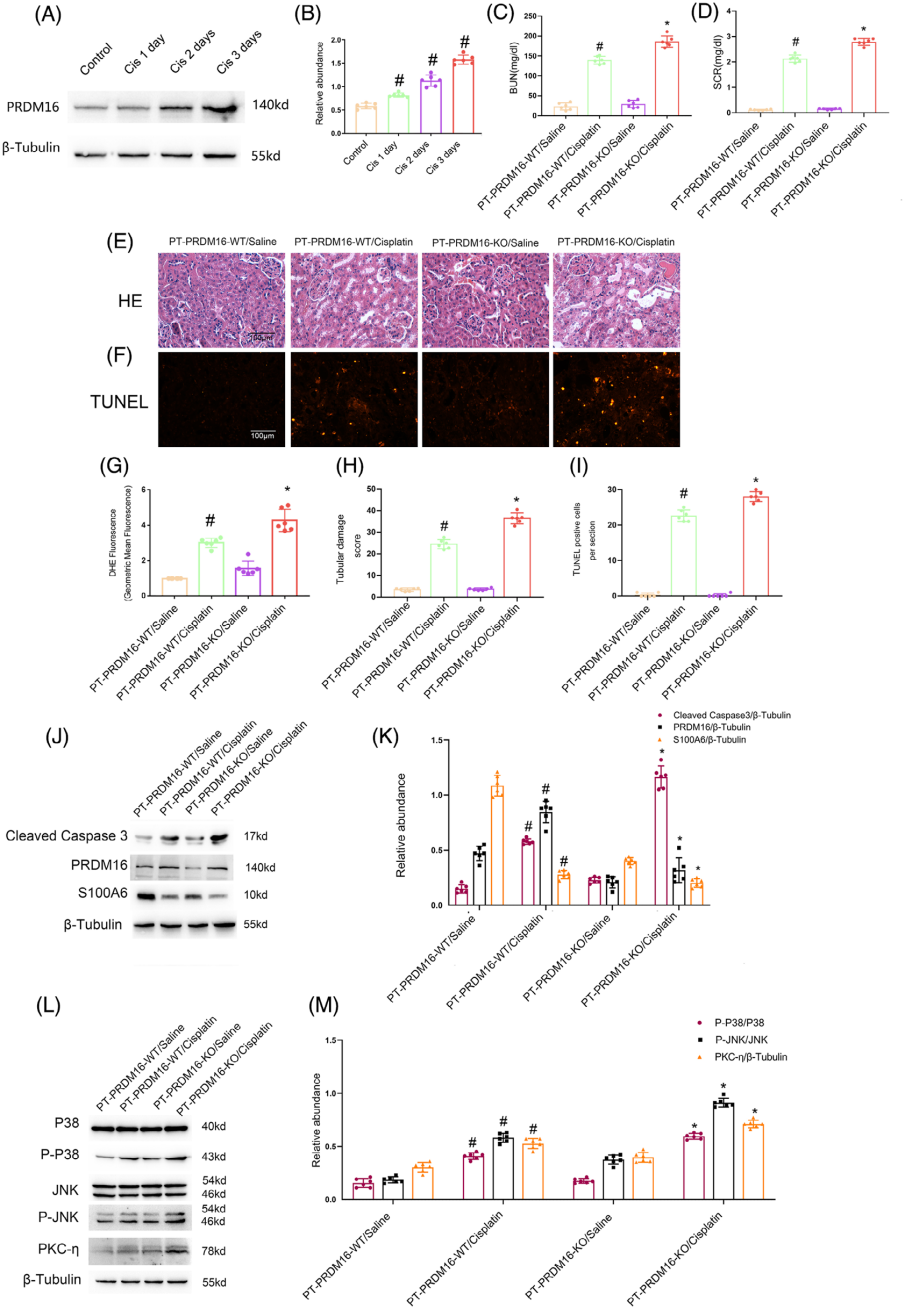
PRDM16‐PT‐KO enhances the cisplatin‐stimulated renal injury, tubular cell apoptosis via regulation S100A6/PKC‐η/ROS/p38MAPK and JNK axis. The bilateral kidney arteries of PRDM16‐PT‐KO (proximal tubule‐specific PRDM16 knockout mouse) and PRDM16‐PT‐WT mice were injected intraperitoneally with cisplatin at 30 mg/kg, and 0.9% saline was used as a control. (A) Immunoblotting of PRDM16, and β‐tubulin. (B) Quantification of protein bands. (C) BUN. (D) Serum creatinine. (E) H&E staining. (F) Representative sections of TUNEL‐positive cells. (G) Quantitative data for DHE (dihydroethidium). (H) Tubular damage scores. (I) The number of TUNEL‐positive cells. (J) Immunoblotting of cleaved‐caspase3, PRDM16, S100A6, and β‐tubulin. (K) Quantification of protein bands. (L) Immunoblotting of p‐P38MAPK, P38MAPK, p‐JNK, JNK, PKC‐η, and β‐tubulin. (M) Quantification of protein bands. Magnification ×400. Scale bar: 100 µM. Means ± SD (*n* = 6). #*p* < 0.05 versus saline. *p < 0.05 versus PRDM16‐PT‐WT plus cisplatin.

To verify the in vitro finding, the proximal PRDM16 deletion mice were used. Before utilizing the tubular PRDM16 deletion mice, we assessed the impact of cisplatin on PRDM16 expression. The immunoblot data indicated that cisplatin could induce PRDM16 expression at the specified time point (Figure 7A,B). PRDM16‐PT‐KO and PRDM16‐PT‐WT mice underwent either I/R (28 min/48 h) or received one intraperitoneal injection of 30 mg/kg cisplatin for 3 days. PRDM16‐PT‐KO mice demonstrated an obvious increase in both I/R and cisplatin treatment‐induced elevation of BUN and Cr levels (Figures 6A,B and 7C,D), along with renal tubular damage (RTD) (Figures 6C,F and 7E,H) and renal tubular cell apoptosis (Figures 6D,G and 7F,I).

To examine the role of PRDM16 in oxidative stress, dihydroethidium (DHE) staining coupled with multicolor FCM was employed.^30^ The DHE analysis results indicated that PRDM16‐PT‐KO noticeably aggravated both I/R‐ and cisplatin‐induced ROS production (Figures 6E and 7G). Immunoblotting results indicated that PRDM16‐PT‐KO mice exhibited enhanced activation of caspase3, p38MAPK, and JNK as well as the expression of PKC‐η via downregulation of S100A6 under basal, I/R, or cisplatin treatment conditions (Figures 6H–K and 7J–M). Taken together, these data show that the PRDM16/S100A6/PKC‐η/ROS/p38MAPK and JNK axes can attenuate AKI in mice induced by I/R and cisplatin.

To confirm the function of PRDM16 in proximal tubular, the proximal PRDM16 overexpression mice were used. PRDM16‐PT‐KI and PRDM16‐PT‐WT mice underwent I/R (28 min/48 h) or received one intraperitoneal injection of 30 mg/kg cisplatin for 3 days. PRDM16‐PT‐KI mice exhibited significantly improved I/R‐ and cisplatin‐triggered increases in BUN and Cr levels (Figures S1 and S2A,B), while reduced RTD (Figures S1 and S2C,F), and renal tubular cell death (Figures S1 and S2D,G). DHE analysis revealed that PRDM16‐PT‐KI mice suppressed both I/R and cisplatin‐induced ROS production (Figures S1E and S2E). The immunoblotting results demonstrated that PRDM16‐PT‐KI mice had suppressed activation of caspase3, p38MAPK, and JNK, as well as reduced expression of PKC‐η via upregulation of S100A6 under basal, I/R, or cisplatin treatment conditions (Figures S1 and S2H–K). Collectively, these data further support that the PRDM16/S100A6/PKC‐η/ROS/p38MAPK and JNK axes play a renoprotective role against the development of I/R‐ and cisplatin‐triggered AKI in mice.

### 2.5 PRDM16 attenuates HK‐2 cell apoptosis mediated by I/R via regulation of the S100A6/PKC‐η/ROS/p38MAPK and JNK signaling pathways

To investigate whether PRDM16 plays a role in human disease and its regulatory mechanisms, we utilized an I/R injury model in HK‐2 cells. First, the DCFH and FCM analysis showed that knockdown of PRDM16 markedly aggravated ROS levels and apoptosis in HK‐2 cells stimulated by I/R injury (Figure S5A–C). The immunoblotting results showed that PRDM16 siRNA1 noticeably improved the activation of cleaved‐caspase3, p38MAPK, and JNK, along with PKC‐η expression under basal and I/R conditions by downregulating S100A6 (Figure S5D–G). Similarly, the immunoblotting results showed that PRDM16 siRNA2 noticeably improved the activation of cleaved‐caspase3, p38MAPK, and JNK, along with PKC‐η expression under basal and I/R conditions by downregulating S100A6 (Figure S5H–K). Conversely, these alterations were mitigated by PRDM16 overexpression (Figure S6A–G). In summary, these findings indicate that PRDM16 serves a protective role against apoptosis in HK‐2 cells stimulated by I/R injury through the regulation of the S100A6/PKC‐η/ROS/p38MAPK and JNK signaling pathways.

### 2.6 S100A6 negatively regulates the I/R‐ and cisplatin‐triggered the progression of AKI

To investigate the function of S100A6 in vivo, C57BL/6 mice were administered AAV2 containing S100A6 shRNA via tail vein injection once, with a follow‐up injection after 3 days. Subsequently, the mice were subjected to either reperfusion or cisplatin injection. Knockdown of S100A6 obviously exacerbated AKI induced by both I/R and cisplatin, as evidenced by increased BUN and Cr levels (Figures S9A–C and S10A,B), along with RTD (Figures S9D,G and S10C,F) and renal tubular cell apoptosis (Figures S9E,H and S10D,G). DHE staining revealed that knockdown of S100A6 remarkably aggravated I/R and cisplatin‐induced ROS production (Figures S9F and S10E). Immunoblotting results further indicated that knockdown of S100A6 obviously enhanced the activation of caspase3, p38MAPK, and JNK, along with the expression of PKC‐η under basal, I/R, or cisplatin treatment conditions (Figures S9I–L and S10H–K). Taken together, the data indicate that knockdown of S100A6 can aggravate AKI in mice following both I/R and cisplatin treatments by activating the PKC‐η/ROS/p38MAPK and JNK axes.

To verify the role of S100A6 in vivo, C57BL/6 mice were administered AAV2 with S100A6 overexpression via tail vein injection once, with a subsequent injection after 3 days. They were then subjected to either reperfusion or cisplatin injection. The overexpression of S100A6 clearly attenuated I/R and cisplatin‐triggered AKI, as evidenced by decreased BUN and Cr levels (Figures S11A–C and S12A,B), along with reduced RTD (Figures S11D,G and S12C,F) and diminished renal tubular cell apoptosis (Figures S11E,H and S12D,G). DHE staining indicated that S100A6 overexpression ameliorated ROS production induced by both I/R and cisplatin (Figures S11F and S12E). The immunoblotting results showed that S100A6 overexpression obviously inhibited the activation of caspase3, p38MAPK, and JNK, as well as the expression of PKC‐η under basal, I/R, or cisplatin treatment conditions (Figures S11I–L and S12H–K). Taken together, these data indicate that S100A6 attenuates AKI in mice induced by I/R or cisplatin via regulation of the PKC‐η/ROS/p38MAPK and JNK axes.

### 2.7 Formononetin protects against apoptosis in BUMPT cell line and mouse kidney via upregulation of PRDM16/S100A6 and inactivation of the PKC‐η/p38MAPK and JNK signaling pathways

To further explore whether formononetin exhibits a protective effect on the BUMPT cellsduring I/R injury, the cells were pretreated with/without formononetin at 20 µM for 30 min, followed by I/R (2 h/2 h). The DCFH assay results indicated that formononetin remarkably decreased I/R‐stimulated ROS production in BUMPT cells (Figure S13A). FCM analysis indicated that formononetin notably reduced I/R‐stimulated BUMPT apoptosis (Figure S13B,C). Immunoblotting further revealed that formononetin markedly suppressed the activation of caspase3, p38MAPK, and JNK, as well as the expression of PKC‐η via the upregulation of PRDM16 and S100A6 under basal and I/R conditions (Figure S13D–G). Taken together, these data show that formononetin suppresses I/R‐stimulated BUMPT apoptosis, along with the activation of p38MAPK and JNK via upregulation of PRDM16/S100A6/PKC‐η/ROS signaling pathways.

To verify the protection function of formononetin in vivo, C57BL/6 mice were exposed to 0/15/25/50 mg/kg formononetin for 3 days. The immunoblot analysis revealed a dose‐dependent response of PRDM16 expression. Specifically, PRDM16 expression showed an increase at 15 mg/kg, reached its peak at 25 mg/kg, and subsequently exhibited a gradual decrease at 50 mg/kg (Figure S14A,B). The mice were also treated with 25 mg/kg formononetin for 0, 3, 7, and 10 days. Immunoblotting data showed that PRDM16 expression was upregulated at days 3 and 7 and peaked at day 10 (Figure S14C,D). Next, the mice were pretreated with 25 mg/kg formononetin for 3 days, followed by I/R (28 min/48 h) or intraperitoneal injection of 30 mg/kg cisplatin for 3 days. Formononetin noticeably ameliorated the I/R and cisplatin‐triggered increase in BUN and Cr levels (Figures S14E,F and S15A,B), as well as RTD (Figures S14G,J and S15C,F) and renal tubular cell apoptosis (Figures S14H,K and S15D,G). DHE staining revealed that formononetin ameliorated I/R and cisplatin‐induced ROS production (Figures S14I and S15E). The immunoblotting data showed that formononetin obviously suppressed the activation of caspase3, p38MAPK, and JNK, as well as the expression of PKC‐η by upregulating PRDM16/S100A6 under basal, I/R or cisplatin treatment conditions (Figures S14L–O and S15H–K). These data indicate that formononetin protects against I/R and cisplatin‐triggered AKI in mice via regulation of the PRDM16/S100A6/PKC‐η/ROS/p38MAPK and JNK axes.

We examined whether formononetin could attenuate I/R‐ or cisplatin‐triggered AKI by upregulating the expression of PRDM16. First, PRDM16‐PT‐KO and PRDM16‐PT‐WT mice were pretreated with/without 25 mg/kg formononetin for 3 days, followed by I/R (28 min/48 h) or one intraperitoneal injection of 30 mg/kg cisplatin for 3 days. Formononetin treatment markedly attenuated the I/R and cisplatin‐triggered increase in BUN and Cr levels (Figures S16 and S17A,B), along with RTD (Figures S16 and S17C,F), ROS production (Figures S16 and S17E), and kidney tubular cell death in PRDM16‐PT‐WT mice (Figures S16 and S17D,G). However, this protective effect was not observed in PRDM16‐PT‐KO mice. The immunoblotting results demonstrated that formononetin remarkably inhibited the activation of caspase3, p38MAPK, and JNK, as well as the expression of PKC‐η via the upregulation of PRDM16 and S100A6 in PRDM16‐PT‐WT mice, but not in PRDM16‐PT‐KO mice, under basal, I/R, or cisplatin treatment conditions (Figures S16 and S17H–K). In summary, these findings imply that the protective effect of formononetin against AKI stimulated by I/R and cisplatin is dependent on PRDM16 expression.

### 2.8 The roles of PRDM16/S100A6/PKC‐η/ROS/P38 MAPK and JNK axes in the human kidney

Although our data indicated that PRDM16 was induced in BUMPT cell line and mouse kidneys (Figure 1), the expression of PRDM16/S100A6/PKC‐η/P38 MAPK and the JNK axis in humans remained unknown. HE and TUNEL staining demonstrated that tubule injury and kidney cell apoptosis were stimulated by I/R (Figure S18A,E,D,H). Immunohistochemistry staining further indicated an increase in PRDM16 and a decrease in S100A6 due to I/R (Figure S18B,F,C,G). The immunoblotting data showed that I/R led to the activation of caspase3, P38, and JNK, as well as the expression of PKC‐η, upregulated PRDM16, and downregulated S100A6 in the patients (Figure S18I–L). When combined with the previous results, these data show that PRDM16/S100A6/ROS/P38 and the JNK axis are responsible for the progression of AKI stimulated by I/R.

## 3 DISCUSSION

Inhibition of renal cell death proves advantageous in mitigating the progression of AKI.^4^, ^5^, ^6^ This research reveals, for the first time, that PRDM16 is induced in the BUMPT cells and mouse kidneys during ischemic injury. Functionally, PRDM16 exhibits a protective effect against renal cell apoptosis, ultimately attenuating I/R‐ and cisplatin‐triggered AKI. Mechanistically, PRDM16 promotes the expression of and interaction with S100A6, thereby impeding the activation of PKC‐η/ROS/p38MAPK and JNK axes (Figure S23). Additionally, we found that formononetin, a small molecule compound, ameliorated the progression of I/R‐ and cisplatin‐triggered AKI by regulating the PRDM16/S100A6/PKC‐η/ROS/p38MAPK and JNK axes.

The role of PRDM16 in cell death remains controversial. While one study suggests that PRDM16 promotes apoptosis in PRDM16,^31^, ^32^ several studies indicate its antiapoptotic function in progenitor cells, prostatic cancer cells, and hematopoietic stem cells.^17^, ^21^, ^22^ Our findings align with the latter perspective, demonstrating PRDM16 as an inhibitor of apoptosis during I/R and cisplatin‐triggered AKI.

Nevertheless, the role of S100A6 in apoptosis is still enigmatic. Although two studies assert that S100A6 induces apoptosis in Calu‐6 lung cancer cells and chondrocytes,^33^, ^34^ several others posit its antiapoptotic effect in cardiomyocytes and clear cell renal cell carcinoma.^32^, ^35^, ^36^, ^37^ In this research, we demonstrate, for the first time, that S100A6 has an antiapoptotic role in renal tubular cells, which is strongly supported by the following evidence. First, knockdown of S100A6 promotes BUMPT cells apoptosis during I/R injury (Figure 4). Reciprocally, S100A6 overexpression prevents I/R‐triggered BUMPT cell death (Figure 5). Mechanistically, while one study suggests that S100A6 inhibits cell apoptosis by suppressing p53,^36^, ^38^ our recent investigation indicates that PKC‐η/p38MAPK and ERK1/2 axes modulate the LPS‐stimulated renal cell apoptosis.^33^ However, the mechanism through which PKC‐η activates p38MAPK and ERK1/2 remains elusive. Recent studies indicate that inhibition of PKC isoforms (e.g., PKCα/β/δ/ζ/λ), reduces the secretion of intracellular ROS,^39^, ^40^, ^41^ which can in turn activate MAPK signaling.^42^, ^43^ In our current study, we found that PKC‐η generates ROS, subsequently activating p38MAPK and ERK1/2, which in turn mediates renal cell apoptosis during AKI triggered by sepsis, I/R, or cisplatin.^38^, ^44^, ^45^, ^46^ Additionally, the study demonstrates that S100 inhibits PKC substrate phosphorylation.^42^ Thus, we reveal a novel antiapoptotic mechanism of S100A6 that inhibits the PKC‐η/ROS/p38MAPK and JNK signaling pathway activation. This effect is substantiated as follows. First, inhibition of S100A6 exacerbates I/R injury in the BUMPT cells via activation of the PKC‐η/ROS/the p38MAPK and JNK signaling pathways (Figure 4). Conversely, overexpression of S100A6 deactivates PKC‐η//ROS/the p38MAPK and JNK during ATP depletion injury (Figure 5). Second, overexpression of S100A6 also impedes the activation of PKC‐η/ROS/p38MAPK and JNK in mice with AKI induced by I/R and cisplatin (Figures S11 and S12). Altogether, these data suggest that S100A6 suppresses PKC‐η‐mediated activation of ROS/the p38MAPK and JNK signaling pathways, thereby mitigating renal cell death during AKI progression.

The aforementioned findings prompted us to examine whether PRDM16 directly regulates the expression of S100A6. Prior research has established PRDM16 as one among numerous transcription factors.^47^ In the present study, the ChIP results demonstrated PRDM16 could directly bind to the S100A6 promoter region (Figure 3). Moreover, existing literature suggests that PRDM16 may interact with other proteins to fulfill associated functions.^30^, ^48^, ^49^, ^50^ Intriguingly, our co‐IP results indicated an interaction between PRDM16 and S100A6 (Figure 3), which was further confirmed by IF staining of PRDM16 and S100A6 (Figure S22). Further experiments revealed that S100A6 could interact with a specific region of PRDM16 containing certain amino acids. Subsequently, we observed that S100A6 could mediate the antiapoptotic effect of PRDM16, as supported by the following evidence. First, S100A6 overexpression reversed the effect of PRDM16 knockdown, thus mitigating apoptosis in the BUMPT cells and dampening the PKC‐η/ROS/p38MAPK and JNK signaling pathway activation induced by I/R (Figure S3A–F). Second, overexpression of PRDM16 did not ameliorate the effects of S100A6 knockdown on apoptotic induction or the activation of PKC‐η/ROS/p38MAPK and JNK signaling pathways in the BUMPT cells following I/R (Figure S4A–F). These findings support the notion that PRDM16 promotes and interacts with S100A6, thereby suppressing the activation of the PKC‐η/ROS/p38MAPK and JNK axes.

Formononetin, an extract used in clinical preparations, attenuates nephrotoxicity‐stimulated AKI by regulating organic‐cation‐transporter‐2, p53, and the PPARα/Nrf2/HO‐1/NQO1 pathway.^39^, ^51^, ^52^ Our study demonstrated that formononetin not only ameliorated cisplatin‐stimulated AKI but also attenuated AKI stimulated by I/R (Figures S14 and S15). Furthermore, formononetin reduced the progression of I/R‐ and cisplatin‐triggered AKI in PRDM16‐PT‐WT mice, but not in PRDM16‐PT‐KO mice, via regulation of the S100A6/PKC‐η/ROS/p38MAPK and the JNK axes (Figures S16 and S17). These findings strongly implicate PRDM16 as a crucial target protein for the intervention of formononetin.

In summary, our findings indicate that PRDM16 protects against I/R and cisplatin‐stimulated AKI through a novel self‐protection mechanism. PRDM16 directly promotes and interacts with S100A6, thereby suppressing the activation of the PKC‐η/ROS/p38MAPK and JNK axes. These molecular alterations were corroborated in the kidneys of I/R‐triggered AKI patients (Figure S18). Furthermore, we observe that formononetin may attenuate the progression of I/R‐ and cisplatin‐triggered AKI by regulating the PRDM16/S100A6/PKC‐η/ROS/p38MAPK and JNK signaling pathways. These findings imply that PRDM16 represents a novel therapeutic target for AKI. Additionally, formononetin, a compound that upregulates PRDM16, holds promise as a drug for treating AKI.

However, this study has some shortcomings at this stage, such as the limited clinical trials of formononetin, which currently cannot be used for the treatment of patients with AKI.

## 4 MATERIALS AND METHODS

### 4.1 Antibodies and reagents

Anti‐PRDM16 (WH3357987) antibody was obtained from Invitrogen (USA), and anti‐S100A6 (ab181975) antibody, PKC‐η (ab179524), and RIP3 (ab195117) were supplied by Abcam (UK) and used for immunoblotting. Anti‐S100A6 (SC‐53950) antibody was procured from Santa Cruz Biotechnology Inc. (USA) and was used for immunofluorescence staining. Anti‐cleaved‐caspase3 (9664), p‐p38 MAPK (4511), p38 MAPK (8690), p‐JNK (4668), JNK (9252), HIF‐1a (36169), and HA‐Tag (3724) antibodies were supplied by Cell Signaling Technology (USA). Anti‐βtubulin antibody was procured from Proteintech (USA). All secondary antibodies were supplied by Thermo Fisher Scientific (USA). Anti‐RIP1 and the calcium ionophore were purchased from Sigma‒Aldrich (Shanghai, China). Antimycin A (ab141904) was obtained from Abcam. Full‐length PRDM16, PRDM16‐1‐890, PRDM16‐890‐1178, PRDM16Δ945–949, 957−960, 981−984, PRDM16Δ1089–1092, 1103−1107, PRDM16Δ945–949, 957−960, 981−984, 1089−1092, 1103−1107, and S100A6 plasmids were established by Shenggong Biology Company (Shanghai, China). The siRNAs targeting PRDM16 (si1: 5′‐CCAGTTCCTGCCTAACTTT‐3′; si2: 5′‐TCAAATGTAAATGTCTGGT‐3′) and S100A6 (si1: 5′‐GGCTTTGATCTACAATGAA‐3′; si2: 5′‐CCAGAAGGAGCTCACCATT‐3′) were synthesized by Ruibo Biology Company (Guangzhou, China). DOX and formononetin were purchased from Shenzhen Institutes of Advanced Technology, Chinese Academy of Sciences. Pifithrin‐a was from EMD chemicals (USA).

### 4.2 Construction of the PRDM16‐PT‐KO model

To substantiate the role of tubular PRDM16 in AKI, the PRDM16‐PT‐KO model was generated as described previously.^26^ The PRDM16‐PT‐WT and PRDM16‐PT‐KO mice then underwent I/R treatment (28 min/48 h). Quantitative immunoblotting revealed that the expression of PRDM16 was lower in the renal cortex tissues of PRDM16‐PT‐KO mice than those of PRDM16‐PT‐WT mice under ischemic injury and normal conditions (Figure S7A,B). These results were additionally corroborated by the immunohistochemistry staining results (Figure S7C,D). These data show that tubular PRDM16 was deleted successfully in the PRDM16‐PT‐KO mice.

### 4.3 Establishment of the PRDM16‐PT‐KI model

To substantiate the role of tubular PRDM16 in AKI, the PRDM16‐PT‐KI model was generated as described previously.^26^ The PRDM16‐PT‐WT and PRDM16‐PT‐KI mice then underwent I/R (28 min/48 h) treatment. Quantitative immunoblotting revealed that the expression of PRDM16 was lower in the renal cortex tissues of PRDM16‐PT‐KI mice than those of PRDM16‐PT‐WT mice under ischemic injury and normal conditions (Figure S8A,B). These results were additionally corroborated by the immunohistochemistry staining results (Figure S8C,D). These data indicate that tubular PRDM16 was deleted successfully in PRDM16‐PT‐KI mice.

### 4.4 AKI model construction through I/R and cisplatin

C57BL/6J mice were supplied by Hunan SJA Laboratory Animal Company. Animals with proximal tubule‐specific PRDM16 deletion or PRDM16 knockin were established through crossing PRDM16 (flox/flox) mice or Rosa26^LSL/LSL^ mice (supplied by Shanghai model organisms) with PEPCK‐Cre mice (obtained from the Vanderbilt University School of Medicine, USA).^53^ The mice then underwent ischemia and nephrotoxic AKI. For ischemic AKI, the bilateral renal arteries were clamped for 28 min with reperfusion for 24 or 48 h. The mice's body temperature was kept around 37°C.^54^ For nephrotoxic AKI, each mouse was injected intraperitoneally with 30 mg/kg cisplatin, while 0.9% saline was used as a control. The majority of mice were sacrificed at 24 or 48 h after ischemic injury or 72 h following cisplatin injection to collect blood samples for the examination of serum Cr and BUN levels and to collect kidney tissues for histology and immunoblotting.^54^ In addition, each mouse was given 25 mg/kg formononetin by oral administration for 3 consecutive days or injected with AAV2 containing S100A6 shRNA or S100A6 via renal artery once to observe for 3 days, 0.9% saline or AAV2 control plasmid used as a control, and then underwent ischemia and nephrotoxic AKI.^53^ All experimental protocols adhered to the guidelines established by the Animal Care Ethics Committee of our hospital. All mice were maintained in standard mouse cages with free access to water and a regular rodent diet.

### 4.5 Cell culture, constructs, transfection, and I/R

The cell line stably expressing PRDM16‐RFP was constructed as mentioned previously. Briefly, BUMPT cells was grown in a six‐well plate with DMEM containing 10% FBS and 1% penicillin‒streptomycin (Gibco; 15140163) at 37°C and 5% CO_2_. rtTA (1 µg), PL623 (1 µg), and PB‐TRE‐PRDM16‐RFP (2 µg) underwent transfection with Lipofectamine2000 (Invitrogen) for 24 h. Next, puromycin (2 µg/mL) was introduced for 1 week to facilitate the selection of monoclonal cells with stable PRDM16‐RFP expression. For the ischemic cell model, BUMPT cells were cultured for 2 h in HBSS (HyClone; SH30030.02) with 1.5 µM calcium ionophore (A23187; Sigma) and 10 µM antimycin A (ab141904; Abcam). The HBSS was subsequently refreshed with DMEM for 0, 2, and 4 h during reperfusion. Cell apoptosis was examined using FCM and immunoblotting of cleaved‐caspase3 and caspase3. After that, the plasmids HA‐DOX‐PRDM16‐ full length, HA‐DOX‐PRDM16‐1‐890, HA‐DOX‐PRDM16‐890‐1178, HA‐DOX‐PRDM16Δ945–949, HA‐DOX‐PRDM16Δ957–960, HA‐DOX‐PRDM16Δ981–984, HA‐DOX‐PRDM16Δ1089–1092, HA‐DOX‐PRDM16Δ1103–1107, and the siRNA of PRDM16 and S100A6 were transfected into BUMPT or HK‐2 cells using Lipofectamine2000.

### 4.6 Luciferase reporter assays

The segments of the S100A6 promoter luciferase reporter plasmids (−500‐0, −400‐0, −300‐0, −200‐0, −100‐0) were established by Shenggong Biology Company. Briefly, the cell line stably expressed HA‐PRDM16‐RFP under transfection with S100A6 promoter plasmids in the presence or absence of DOX for 24 h. The PGMLR‐TK luciferase reporter served as a control vector. Luciferase activities were subsequently quantified using a SpectraMax M5 (Molecular Devices, USA), followed by normalization against pGMLR‐TK activities.

### 4.7 AAV vectors

It is to clone foreign genes into a vector containing inverted terminal repeat (ITR)/MCS. The ITR sequences in these vectors supply all the essential cis‐acting elements required for AAV replication/packaging. The recombinant expression plasmid was cotransfected into AAV‐293 cells along with pHelper (which contains adenovirus‐derived genes) and pAAV‐RC. After transfection for 3 days, the recombinant AAV was generated in the packaging cells. Virus particles were extracted from the infected AAV‐293 cells, and the viral supernatant was subsequently concentrated, followed by purification. The titer of the obtained virus was measured using a quantitative PCR method.

### 4.8 AKI patients and sample collection

The protocols for the collection of human kidney samples received approval from the Review Board and Human Genetic Resources of our hospital. After the patient's consent was obtained and signed, kidney biopsy samples that complied with a clinical diagnostic standard were collected from patients with kidney paracancerous tissue (*n* = 6) and ischemic‐induced AKI (*n* = 6). In addition, the studies conformed to the relevant ethical regulations and were performed according to the Declaration of Helsinki principles. Some kidney specimens underwent fixing with paraformaldehyde (4%) and subsequently stained with HE and immunohistochemistry, and the rest were used for immunoblotting analysis.

### 4.9 Immunoprecipitation

Kidney tissue and cell lysates were collected and subsequently underwent IP using anti‐PRDM16, S100A6, and HA antibodies according to the instructions of the IP kit. The mixture was then eluted and analyzed by gel electrophoresis and immunoblotting for PRDM16, S100A6, and HA.

### 4.10 Protein‐binding site prediction

Protein interaction domains between PRDM16 and S100A6 were estimated based on a previously described bioinformatics protocol.^53^ Briefly, we extracted the amino acid sequences of mouse PRDM16 and S100A6 from the UniProt database (https://www.uniprot.org/). For both proteins, our sequence comparison revealed substantial similarity between mouse and human sequences (S100A6, 96.6%; PRDM16, 88.2%). The PDB file of S100A6 with a full‐length sequence (ID, 6ZDY) was extracted from the RCSB‐PDB database (https://www.rcsb.org). Because the PDB file of full‐length PRDM16 is unavailable, the online platform I‐TASSER (https://zhanggroup.org/I‐TASSER/) was used to model the protein structure of PRDM16. Subsequently, Cluspro V2.0 (http://cluspro.bu.edu/) was used to predict the structural interface that mediates the protein interaction between PRDM16 and S100A6, resulting in possible interactions between S100A6 and one of three functional domains of PRDM16 (N‐terminal, central, and C‐terminal domains). Then, three functional domains of PRDM16 were mapped, and cell experiments were conducted to probe the specific domains that mediate the PRDM16–S100A6 interaction. Finally, we visualized structures and generated animations using PyMOL V2.1 (http://www.pymol. org/).

### 4.11 ChIP analysis

ChIP assay was conducted using primary antibodies against the HA‐tag in accordance with the ChIP kit protocol (Millipore, Boston, MA, USA).^55^ Precipitated DNA was examined by PCR using the following primer pairs: S100A6‐1: 5′‐GGAAGTTGAAGACAGAGCCTTAGGG‐3′ (F) and 5′‐TTCCAGAGAA GCAGTTTCCAGCATC‐3′ (R); S100A6‐2: 5′‐AGGATCAGGAGCTGC CAGTGTC‐3′ (F) and 5′‐GCGACCAGGAGATTGACTTCAAGG‐3′ (R); S100A6‐3: 5′‐GCCCAGCGAGCATTCTCAGTTAC‐3′ (F) and 5′‐TTCTCAAGACTCCTCCCCATTACCC‐3′ (R); S100A6‐4: 5′‐AACTCTGGCTTGTGTGC TGAACC‐3′ (F) and 5′‐ACTGCTTCCCCTCCCTCACTTG‐3′ (R). The sequences of S100A6 promoter region were indicated in Table S2.

### 4.12 ROS detection

The ROS assay kit (Meilum, China; MA0219) containing ROSup and fH‐DA was kept at −20°C and used for ROS assessment. The BUMPT cells were exposed to 1 mL of 10 mM/L DCFH‐DA at 37°C for 30 min, followed by three rinses with serum‐free culture medium. The sections were observed by fluorescent microscopy. The emission and excitation wavelengths were 525 and 488 nm, respectively. The intracellular ROS measure of C57BL/6 mice kidney was analyzed according to the protocol of the DHE analysis Kit (Abcam; ab236206).

### 4.13 Flow cytometry

FCM was conducted following the protocol of the FITC‐Annexin‐V‐Apoptosis Detection Kit (BD; 556547). BUMPT or HK‐2 cells subjected to various treatments were collected using trypsin without EDTA, rinsed three times with PBS, stained with FITC for 15 min and PI for 5 min, and then analyzed by FCM. The FCM procedures were executed in accordance with the kit's instructions (BD Biosciences), and the statistics of apoptosis calculate Q2 and Q3 as the proportion of apoptosis.

### 4.14 BUN and Cr measurement

BUN and Cr were measured using the urea nitrogen content (Beijing Boxbio Science & Technology) and Cr (Nanjing Jiancheng Bioengineering Institute, Nanjing, China) assay kits, respectively.

### 4.15 Relative quantitative real‐time PCR

RNA was extracted from BUMPT cell line or kidney tissues using TRIzol reagent (Invitrogen). The RNA was reverse‐transcribed into cDNA following the protocol of the PrimeScript‐RT reagent kit and gDNA‐Eraser (TaKaRa; RR037A) as previously described.^53^, ^54^ The resulting cDNA was employed as a template for PCR amplification with TB‐Green (TaKaRa; RR820A) and a LightCycler 96 (Roche), using the following primers: PRDM16: 5′‐CAGCAACCTCCAGCGTCACATC‐3′ (F) and 5′‐GCGAAGGTCTTGCCACAGTCAG‐3′ (R); S100A6: 5′‐CCATCTTCCACAAGTACTCTGG‐3′ (F) and 5′‐TACTTCCTGATCCTTGTTACGG‐3′ (R). β‐actin: 5′‐GTGCTATGTTGCTCTAGACTTCG‐3′ (F) and 5′‐ATGCCACAGGATTCCATACC‐3′ (R).

### 4.16 HE staining, TUNEL staining, immunohistochemistry, immunofluorescence, and Western blotting

Kidney tissues were paraffin‐embedded and sectioned at 4‐µM thickness for HE staining, TUNEL staining, immunohistochemistry, and immunofluorescence as described previously.^54^ The medulla and the cortex of the kidney were separated according to renal anatomy.^56^ HE staining was used for the histology analyses. The scoring criteria for kidney tubular injury have been described previously.^57^ Kidney cell apoptosis was assessed by TUNEL staining, with the level of apoptosis determined by the percentage of TUNEL‐positive cells in 10−20 microscopic fields/tissue section.^57^ For immunohistochemistry, tissue sections were incubated overnight at 4°C with primary antibodies (PRDM16 at 1:500 or S100A6 at 1:200), followed by a 30‐min incubation with the secondary antibody at 37°C, and subsequently treated with DAB for 5−10 min. For immunofluorescence staining, tissue sections were incubated overnight at 4°C with specific primary antibodies (PRDM16 at 1:500, S100A6 at 1:200), followed by a 1‐h incubation with a secondary fluorescent antibody at 37°C in the dark, and DAPI was added for 3−5 min. Protein lysate from BUMPT cell line or kidney tissues was collected, followed by centrifugation. The supernatants were separated through SDS‐PAGE, transferred onto PVDF membranes, incubated overnight at 4°C with the primary antibody, and then with the secondary antibody for 60 min.

### 4.17 Statistics

All data were expressed as means ± SD. Two‐tailed Student *t*‐tests were utilized for comparisons of two groups, while one‐way ANOVA was employed for comparisons of multiple groups. *p* Values < 0.05 were considered statistically significant.

## AUTHOR CONTRIBUTIONS

Dongshan Zhang conceptualized and planned the experiments. Xiaozhou Li, Fang Xu, and Pan Zhang performed the experiments. Liufeng Mao, Yong Guo, and Huiling Li analyzed the data. Yuxing Xie, Yijian Li, Yingjun Liao, and Junxiang Chen prepared materials and reagents. Donghai Wu was involved in data analysis. Dongshan Zhang drafted the first manuscript, and all authors participated in the review. The final manuscript was read and endorsed by all authors.

## CONFLICT OF INTEREST STATEMENT

The authors declare that they have no conflict of interest.

## ETHICS STATEMENT

This research received approval from the Review Board of our hospital (No. 2020310). We recruited 18 patients, all of whom provided written informed consent before participating. All animal experiments were carried out in compliance with the guidelines set by the Animal Care Ethics Committee of our hospital (No. 2018065).

## Supporting information



Supporting Information

## Data Availability

The datasets used and/or analyzed in the study are available in the supplements or from the corresponding author upon reasonable request.
