# Supplementary material for: Overexpression of PRDM16 attenuates acute kidney injury progression: genetic and pharmacological approaches
Source: MedComm (2020). 2024 Sep 21;5(10):e737. doi: 10.1002/mco2.737 (PMC11416085; doi:10.1002/mco2.737)
Supplement: Supplementary file 1 — Supporting Information [file MCO2-5-e737-s001.pdf]

Overexpression of PRDM16 Attenuates Acute Kidney Injury Progression: Genetic and Pharmacological Approaches

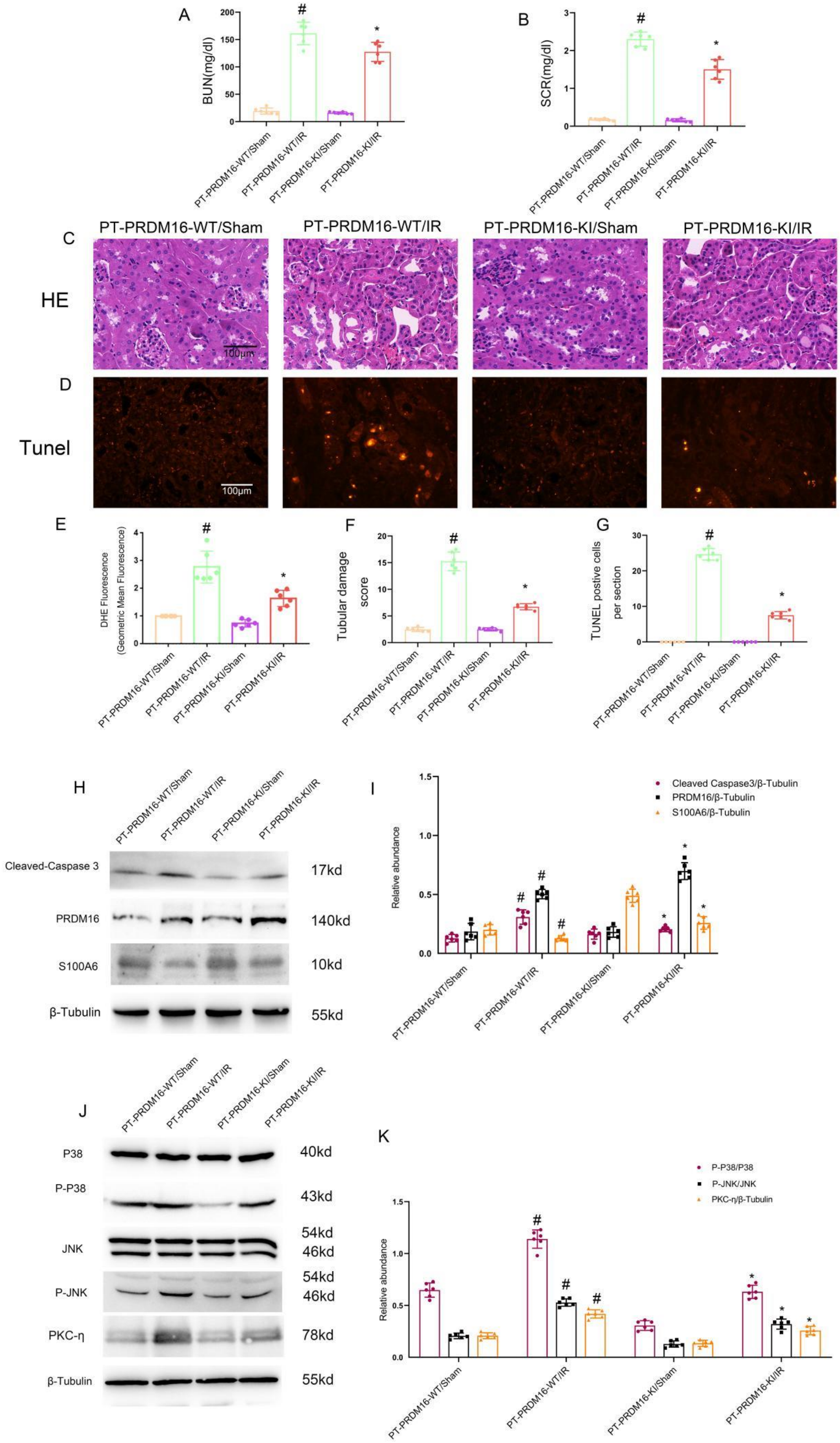
A
BUN(mg/dl)
B
SCR(mg/dl)
PT-PRDM16-WT/Sham
PT-PRDM16-WT/IR
PT-PRDM16-KI/Sham
PT-PRDM16-KI/IR
C
HE
D
Tunel
100μm
E
DHE Fluorescence (Geometric Mean Fluorescence)
F
Tubular damage score
G
TUNEL postive cells per section
H
Cleaved-Caspase 3
PRDM16
S100A6
β-Tubulin
17kd
140kd
10kd
55kd
I
Relative abundance
Cleaved Caspase3/β-Tubulin
PRDM16/β-Tubulin
S100A6/β-Tubulin
J
P38
P-P38
JNK
P-JNK
PKC-η
β-Tubulin
40kd
43kd
54kd
46kd
78kd
55kd
K
P-P38/P38
P-JNK/JNK
PKC-η/β-Tubulin

Figure S1: PT-PRDM16-KI ameliorated the IR-induced renal injury, tubular cell apoptosis via regulation S100A6/ PKC-η/ ROS/p38MAPK and JNK axis.

The bilateral renal arteries of PT-PRDM16-KI (proximal tubule-specific PRDM16 knock-in) and PT-PRDM16-WT littermate mice were clamped for 28min and then released for 48h to establish an I/R (Ischemic Reperfusion) model. (A) BUN (Blood Urea Nitrogen). (B) Serum creatinine. (C) H&E (Hematoxylin and eosin staining). (D) Representative sections of TUNEL-positive cells. (E) Quantitative data for DHE (dihydroethidium). (F) Tubular damage score. (G) The number of TUNEL-positive cells. (H)The immunoblot analysis of Cleaved Caspase3, PRDM16, S100A6, and β-tubulin. (I) Densitometry analysis of immunoblot bands. (J)The immunoblot analysis of P-P38MAPK, P38MAPK, P-JNK, JNK, PKC-η, and β-tubulin. (K) Densitometry analysis of immunoblot bands. Original magnification x 400. Scale Bar:100μM. Data are expressed as means ± SD (n=6). # $P<0.05$ versus sham group. * $P<0.05$ versus PT-PRDM16 WT with IR group.

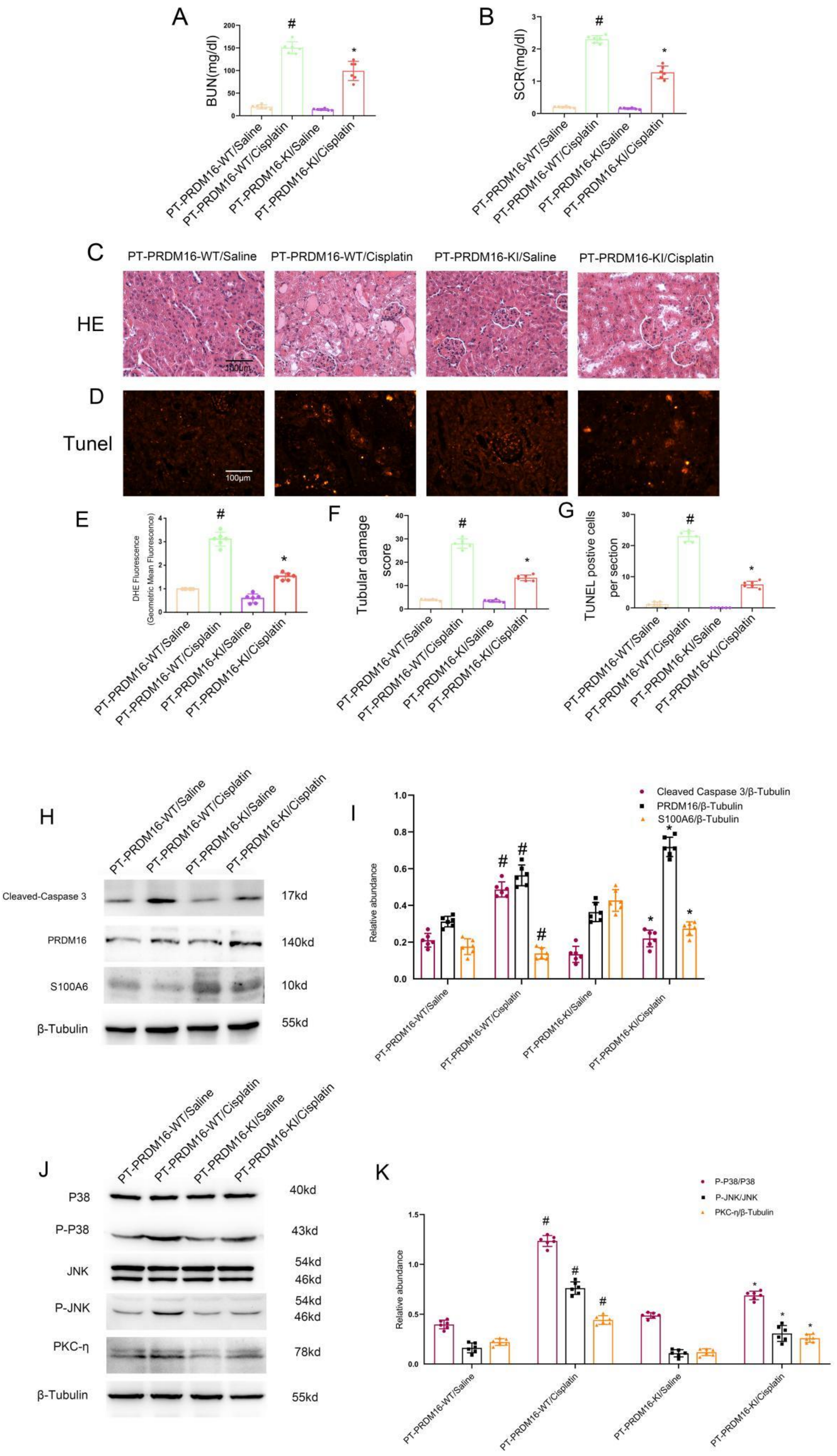
A
B
BUN(mg/dl)
SCR(mg/dl)
PT-PRDM16-WT/Saline
PT-PRDM16-WT/Cisplatin
PT-PRDM16-KI/Saline
PT-PRDM16-KI/Cisplatin
C
D
HE
Tunel
100μm
E
F
G
DHE Fluorescence (Geometric Mean Fluorescence)
Tubular damage score
TUNEL postive cells per section
H
I
Cleaved-Caspase 3
PRDM16
S100A6
β-Tubulin
17kd
140kd
10kd
55kd
Cleaved Caspase 3/β-Tubulin
PRDM16/β-Tubulin
S100A6/β-Tubulin
Relative abundance
J
K
P38
P-P38
JNK
P-JNK
PKC-η
40kd
43kd
54kd
46kd
78kd
P-P38/P38
P-JNK/JNK
PKC-η/β-Tubulin

Figure S2: PT-PRDM16-KI ameliorated the Cisplatin-induced renal injury, tubular cell apoptosis via regulation S100A6/PKC-η/ROS/p38MAPK and JNK axis.

The bilateral renal arteries of PT-PRDM16-KI (proximal tubule-specific PRDM16 knock-in) and PT-PRDM16-WT littermate mice were injected intraperitoneally with cisplatin at 30 mg/kg, and 0.9% saline was used as a control. (A) BUN (Blood Urea Nitrogen). (B) Serum creatinine. (C) H&E (Hematoxylin and eosin staining). (D) Representative sections of TUNEL-positive cells. (E) Quantitative data for DHE (dihydroethidium). (F) Tubular damage score. (G)The number of TUNEL-positive cells. (H)The immunoblot analysis of Cleaved Caspase3, PRDM16, S100A6, and β-tubulin. (I) Densitometry analysis of immunoblot bands. (J)The immunoblot analysis of P-P38MAPK, P38MAPK, P-JNK, JNK, PKC-η, and β-tubulin. (K)Densitometry analysis of immunoblot bands. Original magnification x 400. Scale Bar:100μM. Data are expressed as means ± SD (n=6). # *P<0.05* versus saline group. * *P<0.05* versus PT-PRDM16 WT with Cisplatin group.

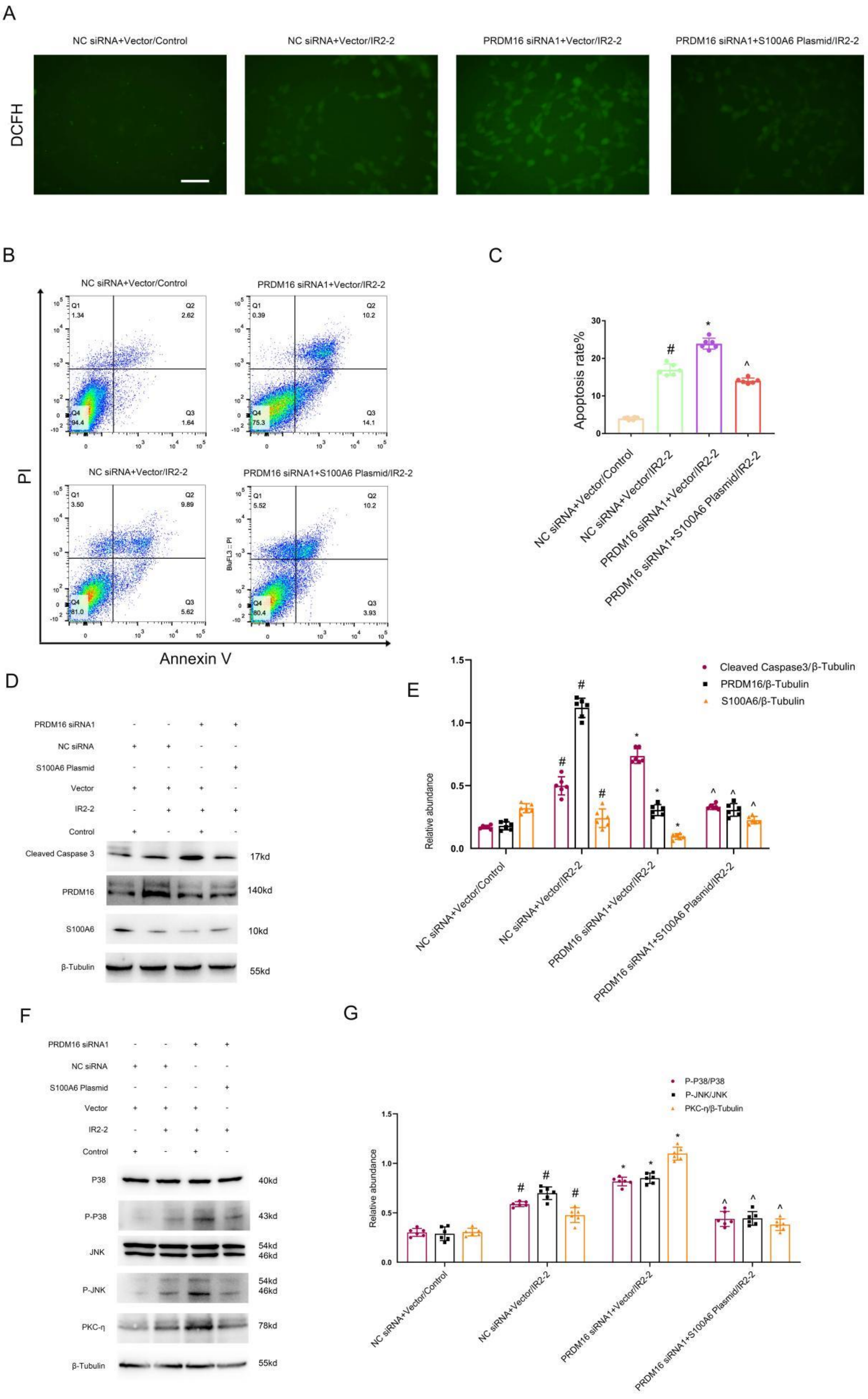
A
NC siRNA+Vector/Control
NC siRNA+Vector/IR2-2
PRDM16 siRNA1+Vector/IR2-2
PRDM16 siRNA1+S100A6 Plasmid/IR2-2
DCFH
B
NC siRNA+Vector/Control
PRDM16 siRNA1+Vector/IR2-2
NC siRNA+Vector/IR2-2
PRDM16 siRNA1+S100A6 Plasmid/IR2-2
PI
Annexin V
C
Apoptosis rate%
D
PRDM16 siRNA1
NC siRNA
S100A6 Plasmid
Vector
IR2-2
Control
Cleaved Caspase 3
PRDM16
S100A6
β-Tubulin
17kd
140kd
10kd
55kd
E
Cleaved Caspase3/β-Tubulin
PRDM16/β-Tubulin
S100A6/β-Tubulin
Relative abundance
F
P38
P-P38
JNK
P-JNK
PKC-η
40kd
43kd
54kd
46kd
78kd
55kd
G
P-P38/P38
P-JNK/JNK
PKC-η/β-Tubulin
Relative abundance

Figure S3:The PRDM16 siRNA with or without S100A6 plasmid was transfected into BUMPT cells, and then subjected to I(2h)/R(2h) treatment.

(A)ROS(reactive oxygen species) level assessed by DCFH(dichlorodihydrofluorescein). (B) Flow cytometry analysis. (C) quantitative data for apoptosis. (D)The immunoblot analysis of Cleaved Caspase3, PRDM16, S100A6, and β-tubulin. (E) Densitometry analysis of immunoblot bands. (F)The immunoblot analysis of P-P38MAPK, P38MAPK, P-JNK, JNK, PKC-η, and β-tubulin. (G) Densitometry analysis of immunoblot bands. Original magnification x 400. Scale Bar:100μM. Data are expressed as means ±SD (n=6). # $P<0.05$ versus scramble with Control group. * $P<0.05$ versus scramble with IR group. ^$P<0.05$ versus PRDM16 siRNA with IR group.

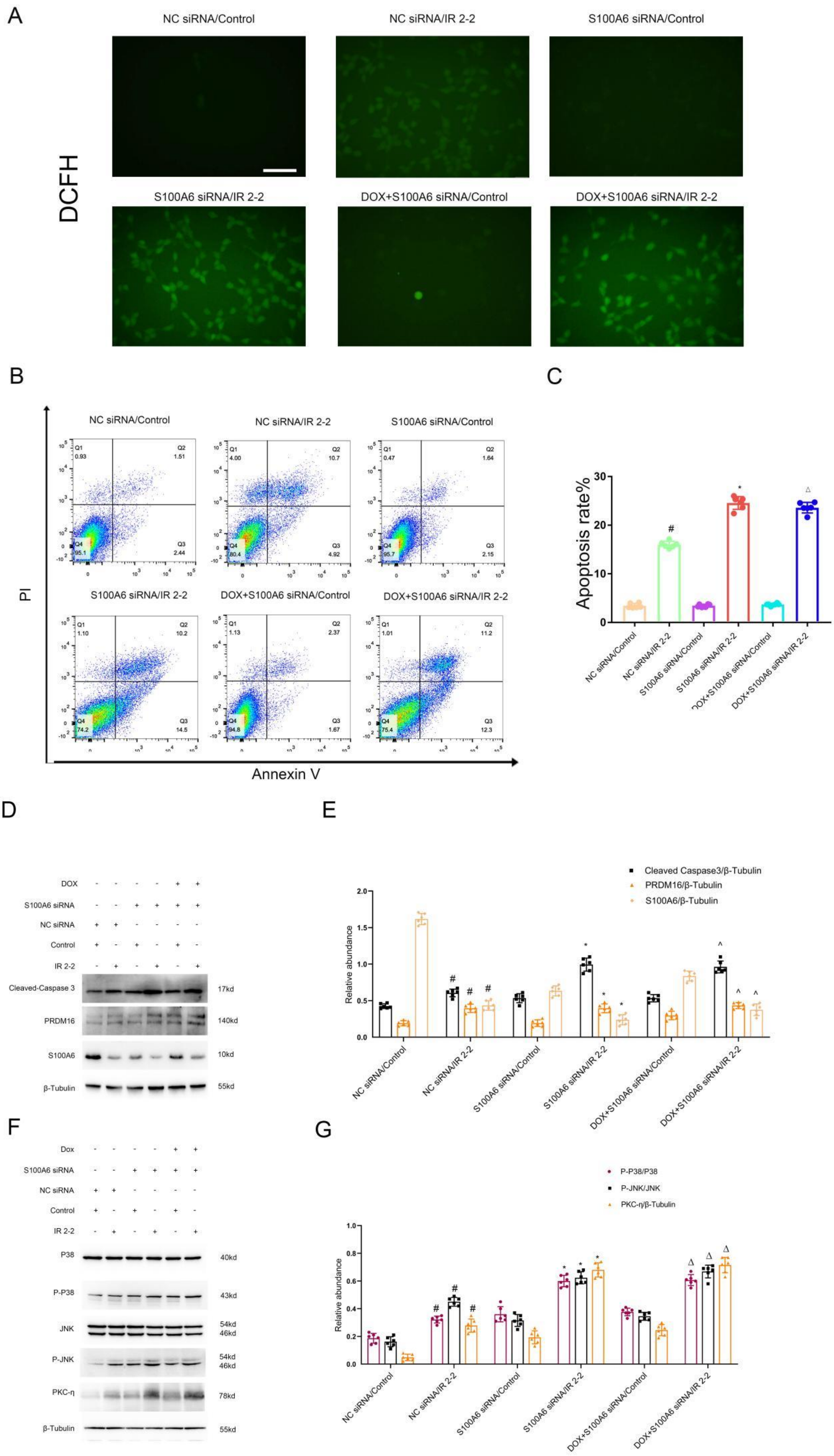
A
NC siRNA/Control
NC siRNA/IR 2-2
S100A6 siRNA/Control
DCFH
S100A6 siRNA/IR 2-2
DOX+S100A6 siRNA/Control
DOX+S100A6 siRNA/IR 2-2
B
C
PI
Annexin V
Apoptosis rate%
D
E
DOX
S100A6 siRNA
NC siRNA
Control
IR 2-2
Cleaved-Caspase 3
PRDM16
S100A6
β-Tubulin
17kd
140kd
10kd
55kd
Cleaved Caspase3/β-Tubulin
PRDM16/β-Tubulin
S100A6/β-Tubulin
Relative abundance
F
G
Dox
P38
P-P38
JNK
P-JNK
PKC-η
40kd
43kd
54kd
46kd
78kd
P-P38/P38
P-JNK/JNK
PKC-η/β-Tubulin

Figure S4: Knockdown of S100A6 enhanced IR-induced apoptosis in BUMPT cells was not attenuated by the overexpression of PRDM16.

The siRNA of S100A6 was transfected into the PRDM16-RFP stably expressed cell line plus with or without DOX treatment and then exposed to ischemic for 2 hours and recovery for 2 hours. **(A)** ROS (reactive oxygen species) level assessed by DCFH(dichlorodihydrofluorescein). (B) Flow cytometry analysis. (C) quantitative data for apoptosis. (D)The immunoblot analysis of Cleaved Caspase3, PRDM16, S100A6, and β-tubulin. (E) Densitometry analysis of immunoblot bands. (F)The immunoblot analysis of P-P38MAPK, P38MAPK, P-JNK, JNK, PKC-η, and β-tubulin. (G) Densitometry analysis of immunoblot bands. Original magnification x 400. Scale Bar:100μM. Data are expressed as means ± SD (n=6). # P<0.05 versus Control group. * P<0.05 versus IR group. ∆P>0.05 versus IR with S100A6 siRNA group.

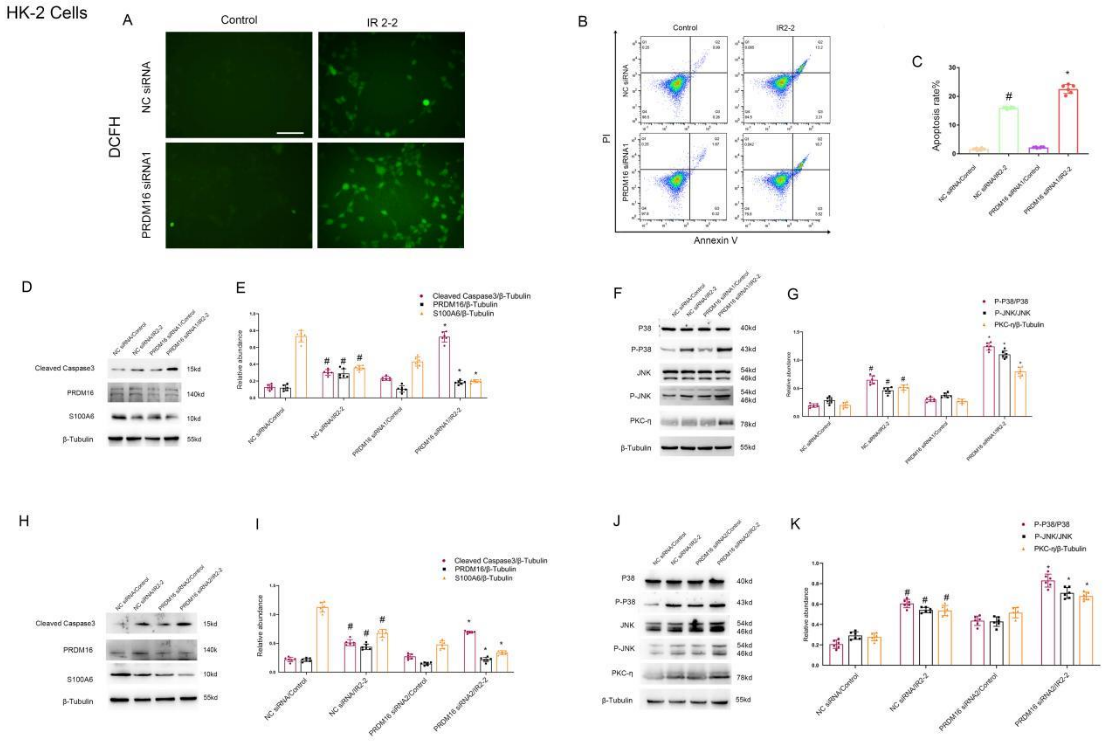

Figure S5: Knockdown of PRDM16 aggravate the I/R-apoptosis in HK-2 cells

The PRDM16 siRNA was transfected into HK-2 cells and then exposed to for 2 hours and recovery for 2 hours. **(A)** ROS (reactive oxygen species) level assessed by DCFH (dichlorodihydrofluorescein). (B) Flow cytometry analysis. (C) quantitative data for apoptosis. (D)The immunoblot analysis of Cleaved Caspase3, PRDM16, S100A6, and β-tubulin. (E) Densitometry analysis of immunoblot bands. (F)The immunoblot analysis of p-P38MAPK, P38MAPK, p-JNK, JNK, PKC-η, and β-tubulin. (G) Densitometry analysis of immunoblot bands. (H)The immunoblot analysis of cleaved Caspase3, PRDM16, S100A6, and β-tubulin. (I) Densitometry analysis of immunoblot bands. (J)The immunoblot analysis of P-P38MAPK, P38MAPK, P-JNK, JNK, PKC-η, and β-tubulin. (K) Densitometry analysis of immunoblot bands. Data are expressed as means ±SD(n=6). # $P<0.05$ versus scramble with Control group. * $P<0.05$ versus scramble with IR group.

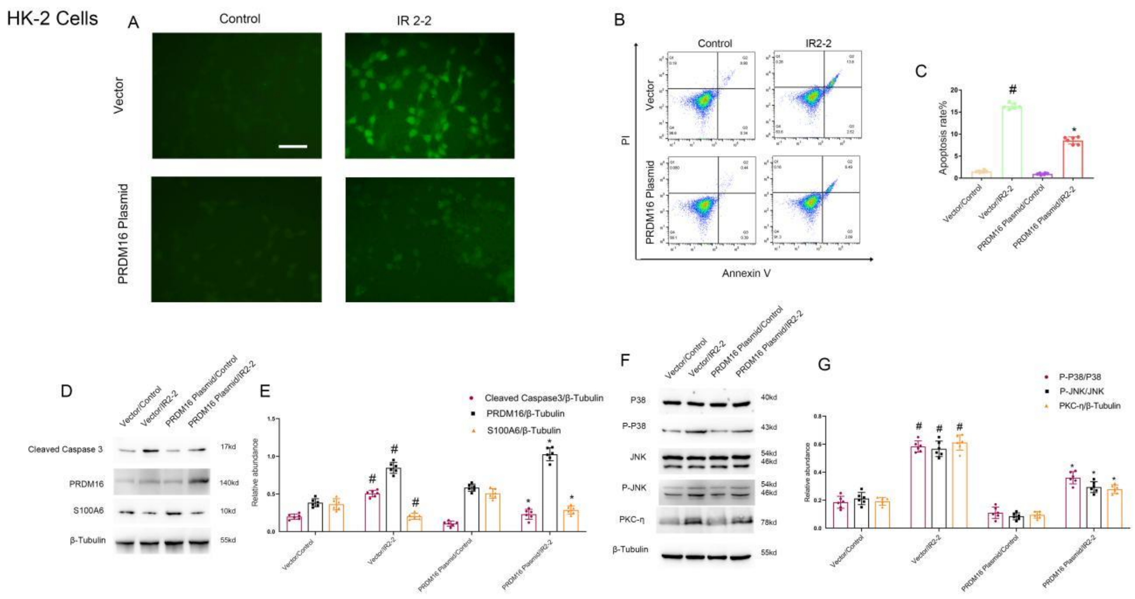

Figure S6: Overexpression of PRDM16 extenuated the I/R-apoptosisinHK-2 cells

The PRDM16 plasmid was transfected into HK-2 cells and then exposed to ischemic

for 2 hours and recovery for 2 hours. **(A)** ROS (reactive oxygen species) level assessed by DCFH(dichlorodihydrofluorescein). (B) Flow cytometry analysis. (C) quantitative data for apoptosis. (D)The immunoblot analysis of Cleaved Caspase3, PRDM16, S100A6, and β-tubulin. (E) Densitometry analysis of immunoblot bands. (F)The immunoblot analysis of P-P38MAPK, P38MAPK, P-JNK, JNK, PKC-η, and β-tubulin. (G) Densitometry analysis of immunoblot bands. Original magnification x 400. Scale Bar:100μM. Data are expressed as means ± SD (n=6). # *P<0.05* versus scramble with Control group. * *P<0.05* versus scramble with IR group.

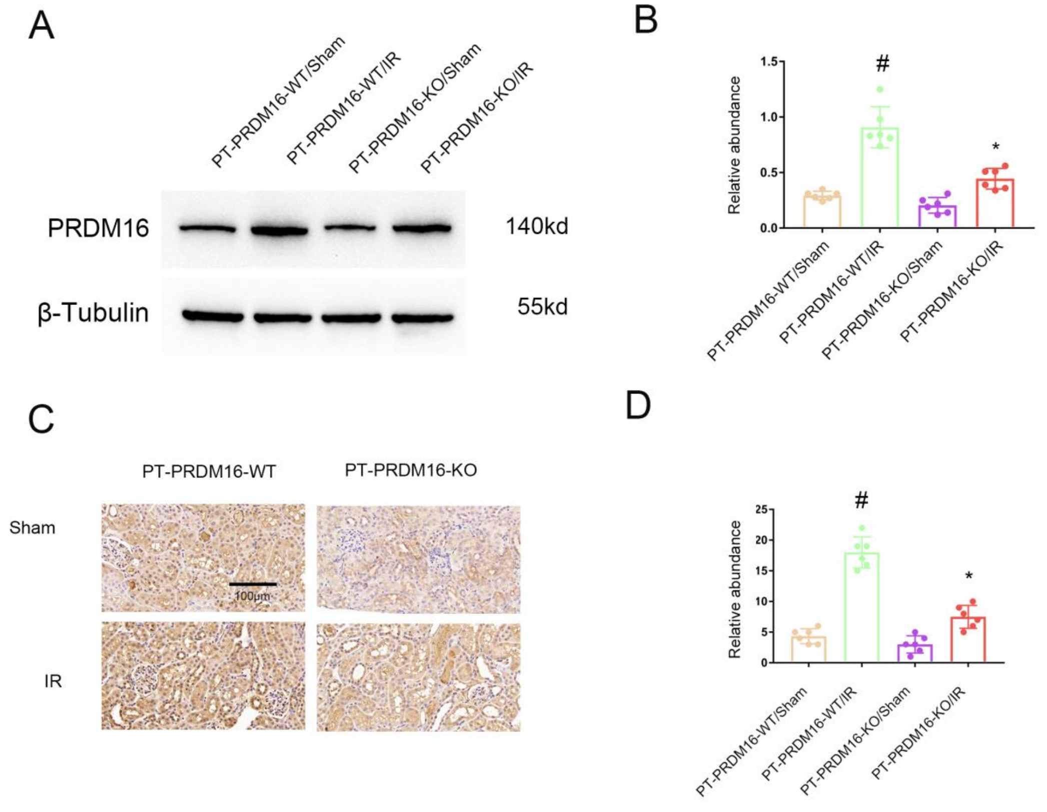

Figure S7: Creation and characteristic of the PT-PRDM16-KO mouse model.

(A) The immunoblots analysis of the expression of PRDM16 in cortex of kidney. (B) Densitometry analysis of immunoblot bands. (C)immunohistochemical staining of the expression of PRDM16. (D) Quantification analysis of PRDM16 staining. Original magnification x 400. Scale Bar:100μM. Data are expressed as mean ± SD (n = 6). # *P<0.05* versus sham group. * *P<0.05* versus PT-PRDM16-WT with IR group.

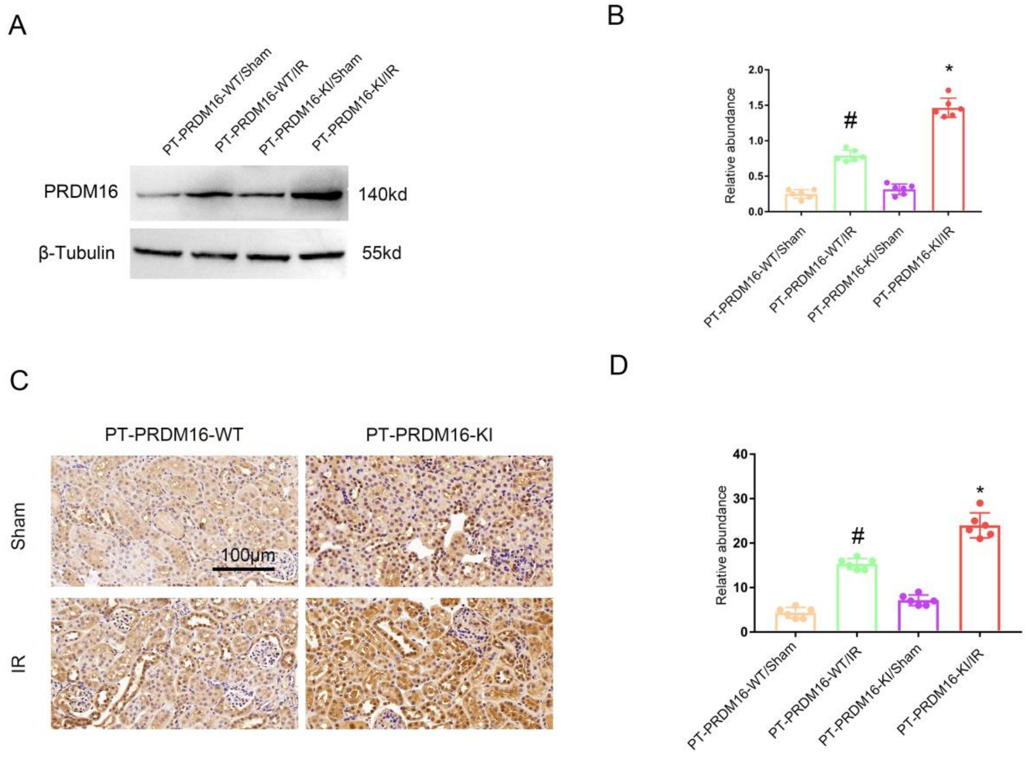

Figure S8: Creation and characteristic of the PT-PRDM16-KI mouse model.

(A) The immunoblots analysis of the expression of PRDM16 and β-tubulin. (B) Densitometry analysis of immunoblot bands. (C) immunohistochemical staining of the expression of PRDM16. (D) Quantification analysis of PRDM16 staining. Original magnification x 400. Scale Bar:100μM. Data are expressed as mean ± SD (n = 6). # *P<0.05* versus sham group. * *P<0.05* versus PT-PRDM16-WT with IR group.

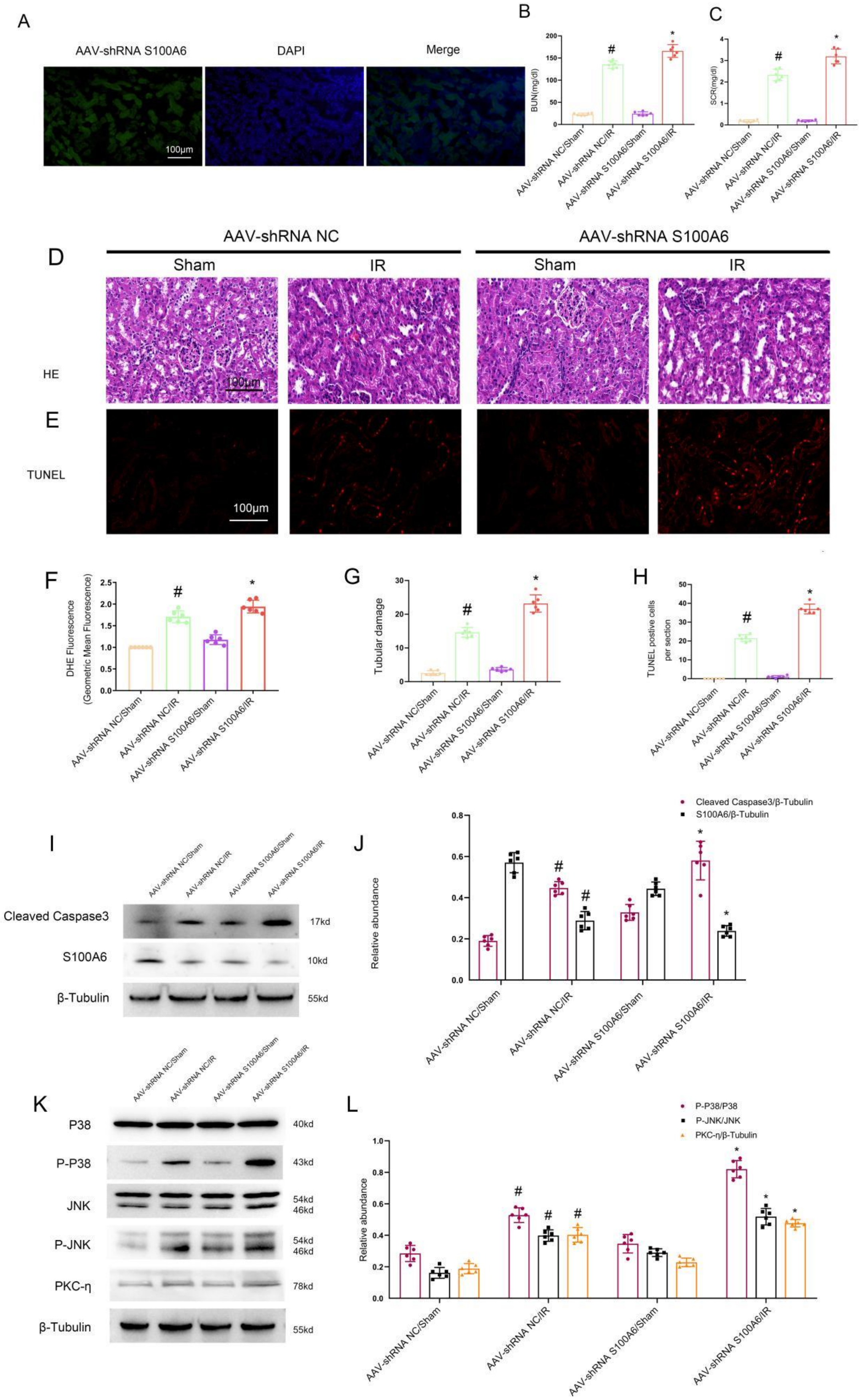

A
AAV-shRNA S100A6
DAPI
Merge
100μm
B
BUN(mg/dl)
C
SCR(mg/dl)
AAV-shRNA NC/Sham
AAV-shRNA NC/IR
AAV-shRNA S100A6/Sham
AAV-shRNA S100A6/IR
D
AAV-shRNA NC
AAV-shRNA S100A6
Sham
IR
HE
E
TUNEL
F
DHE Fluorescence (Geometric Mean Fluorescence)
G
Tubular damage
H
TUNEL postive cells per section
I
Cleaved Caspase3
S100A6
β-Tubulin
17kd
10kd
55kd
J
Cleaved Caspase3/β-Tubulin
S100A6/β-Tubulin
Relative abundance
K
P38
P-P38
JNK
P-JNK
PKC-η
40kd
43kd
54kd
46kd
78kd
L
P-P38/P38
P-JNK/JNK
PKC-η/β-Tubulin

Figure S9: Knockdown of S100A6enhances IR-induced renal injury and tubular cell apoptosis via activation of the PKC-η/ROS/p38MAPK and JNK axes.

TheC57BL/6 mice were injected with adeno-associated virus 2 (AAV2) carrying of S100A6 shRNA via renal artery once, after 3 days, and then subjected to I(28min)/R(48h). (A) Immunofluorescence of AAV-shRNA S100A6 (B) BUN. (C) Serum creatinine. (D) Hematoxylin and eosin staining. (E) Representative sections of TUNEL-positive cells. (F) Quantitative data for DHE (dihydroethidium). Representative sections of TUNEL-positive cells. (G) Tubular damage score. (H) The number of TUNEL-positive cells. (I)Immunoblot analysis of cleaved caspase-3, PRDM16, S100A6, and β-tubulin. (J) Densitometry analysis of immunoblot bands. (K) Immunoblot analysis of P-P38MAPK, P38MAPK, P-JNK, JNK, PKC-η, and β-tubulin. (L) Densitometry analysis of immunoblot bands. Original magnification x 400. Scale bar: 100 µM. Data are expressed as the means ± SDs (n=6). # $P<0.05$ versus sham group. * $P<0.05$ versus IR group.

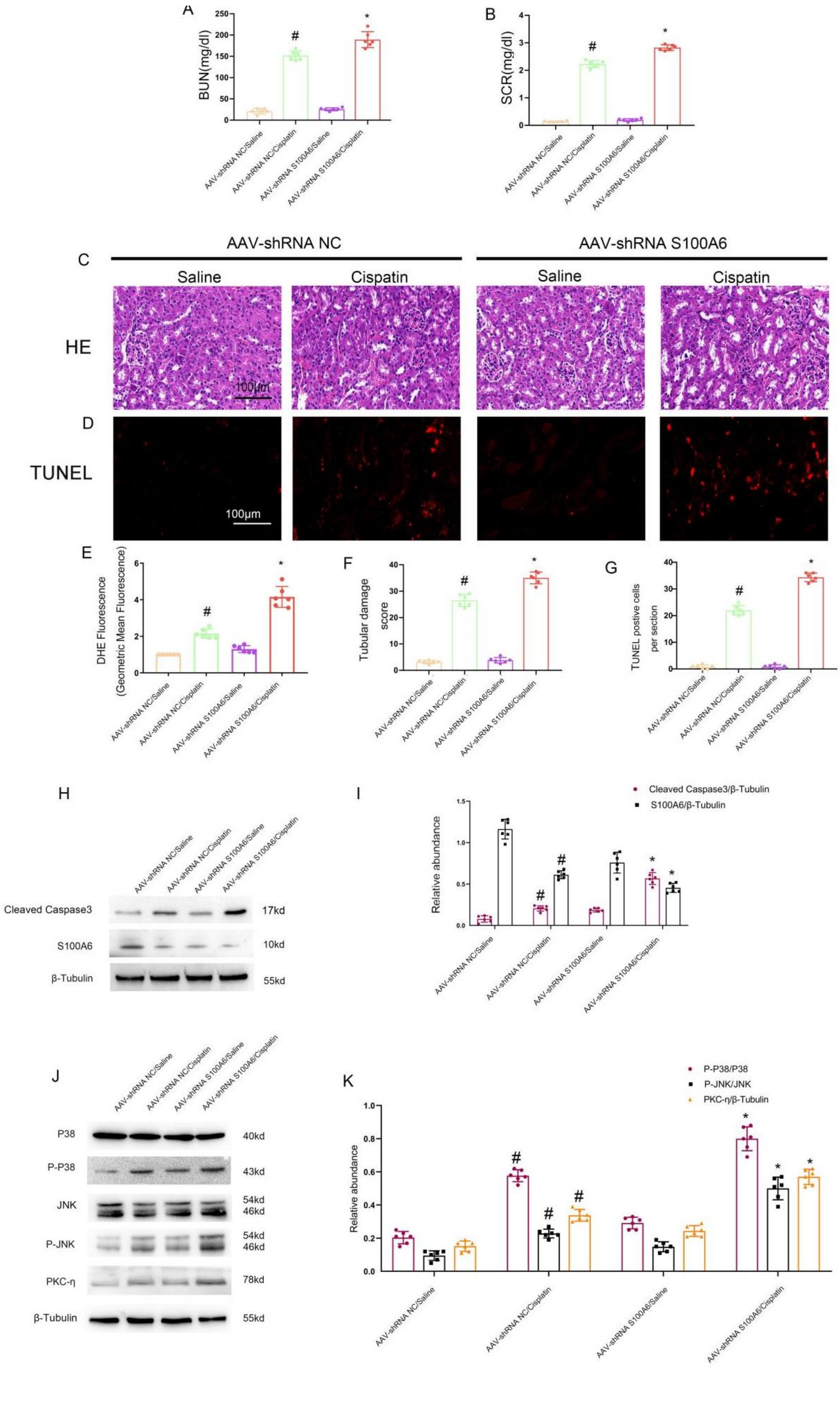
A
BUN(mg/dl)
AAV-shRNA NC/Saline
AAV-shRNA NC/Cisplatin
AAV-shRNA S100A6/Saline
AAV-shRNA S100A6/Cisplatin
B
SCR(mg/dl)
AAV-shRNA NC
AAV-shRNA S100A6
C
Saline
Cispatin
HE
100μm
D
TUNEL
E
DHE Fluorescence (Geometric Mean Fluorescence)
F
Tubular damage score
G
TUNEL postive cells per section
H
Cleaved Caspase3
S100A6
β-Tubulin
17kd
10kd
55kd
I
Cleaved Caspase3/β-Tubulin
S100A6/β-Tubulin
Relative abundance
J
P38
P-P38
JNK
P-JNK
PKC-η
40kd
43kd
54kd
46kd
78kd
K
P-P38/P38
P-JNK/JNK
PKC-η/β-Tubulin

Figure S10: Knockdown of S100A6 enhances cisplatin-induced renal injury and tubular cell apoptosis by regulating the S100A6/PKC-η/ ROS/p38MAPK and JNK axes.

The C57BL/6 mice were injected with adeno-associated virus 2 (AAV2) carrying of S100A6 shRNA via renal artery once, after 3 days, and then injected intraperitoneally with cisplatin at 30 mg/kg, 0.9% saline was used as a control. (A) BUN. (B) Serum creatinine. (C) Hematoxylin and eosin staining. (D) Representative sections of TUNEL-positive cells. (E) Quantitative data for DHE(dihydroethidium). (F) Tubular damage score. (G) The number of TUNEL-positive cells. (H)Immunoblot analysis of cleaved caspase-3, PRDM16, S100A6, and β-tubulin. (I) Densitometry analysis of immunoblot bands. (J) Immunoblot analysis of P-P38MAPK, P38MAPK, P-JNK, JNK, PKC-η, and β-tubulin. (K) Densitometry analysis of immunoblot bands. Original magnification x 400. Scale bar: 100 μM. Data are expressed as the means ± SDs (n=6). # $P<0.05$ versus the saline group. * $P<0.05$ versus cisplatin group.

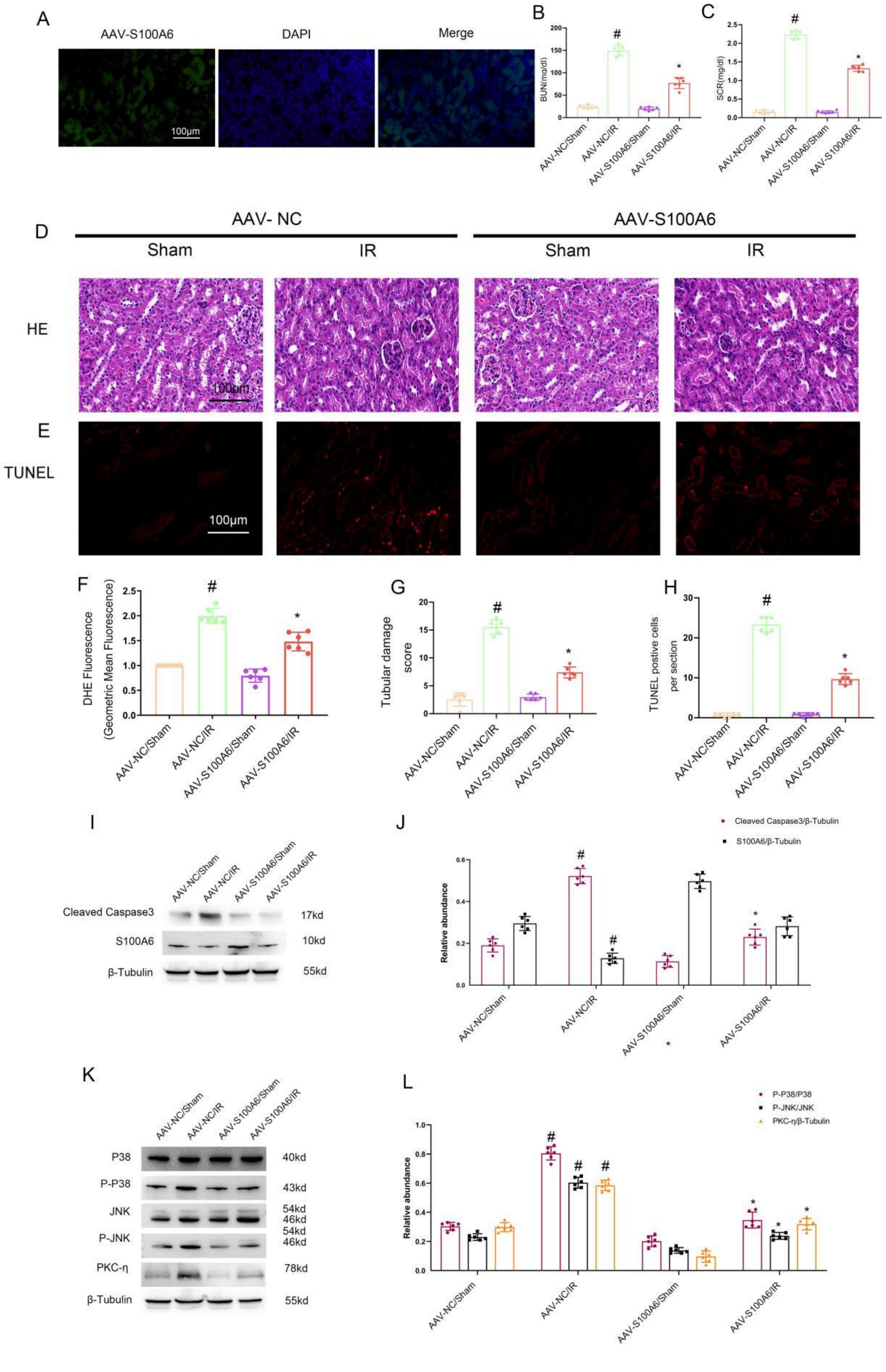

Figure S11: S100A6 plasmids attenuate IR-induced renal injury and tubular cell

apoptosis via inactivation ofthe PKC-η/ ROS/p38MAPK and JNK axes.

The C57BL/6 mice were injected with adeno-associated virus 2 (AAV2) carrying of S100A6 via renal artery once, after 3 days, and then subjected to I(28min)/R(48h). (A) Immunofluorescence of AAV-S100A6. (B) BUN. (C) Serum creatinine. (D) Hematoxylin and eosin staining. (E) Representative sections of TUNEL-positive cells. (F) Quantitative data for DHE(dihydroethidium). (G) Tubular damage score. (H) The number of TUNEL-positive cells. (I)Immunoblot analysis of cleaved caspase-3, PRDM16, S100A6, and β-tubulin. (J) Densitometry analysis of immunoblot bands. (K) Immunoblot analysis of P-P38MAPK, P38MAPK, P-JNK, JNK, PKC-η, and β-tubulin. (L) Densitometry analysis of immunoblot bands. Original magnification x 400. Scale bar: 100 μM. Data are expressed as the means ± SDs (n=6). # $P<0.05$ versus sham group. * $P<0.05$ versus IR group.

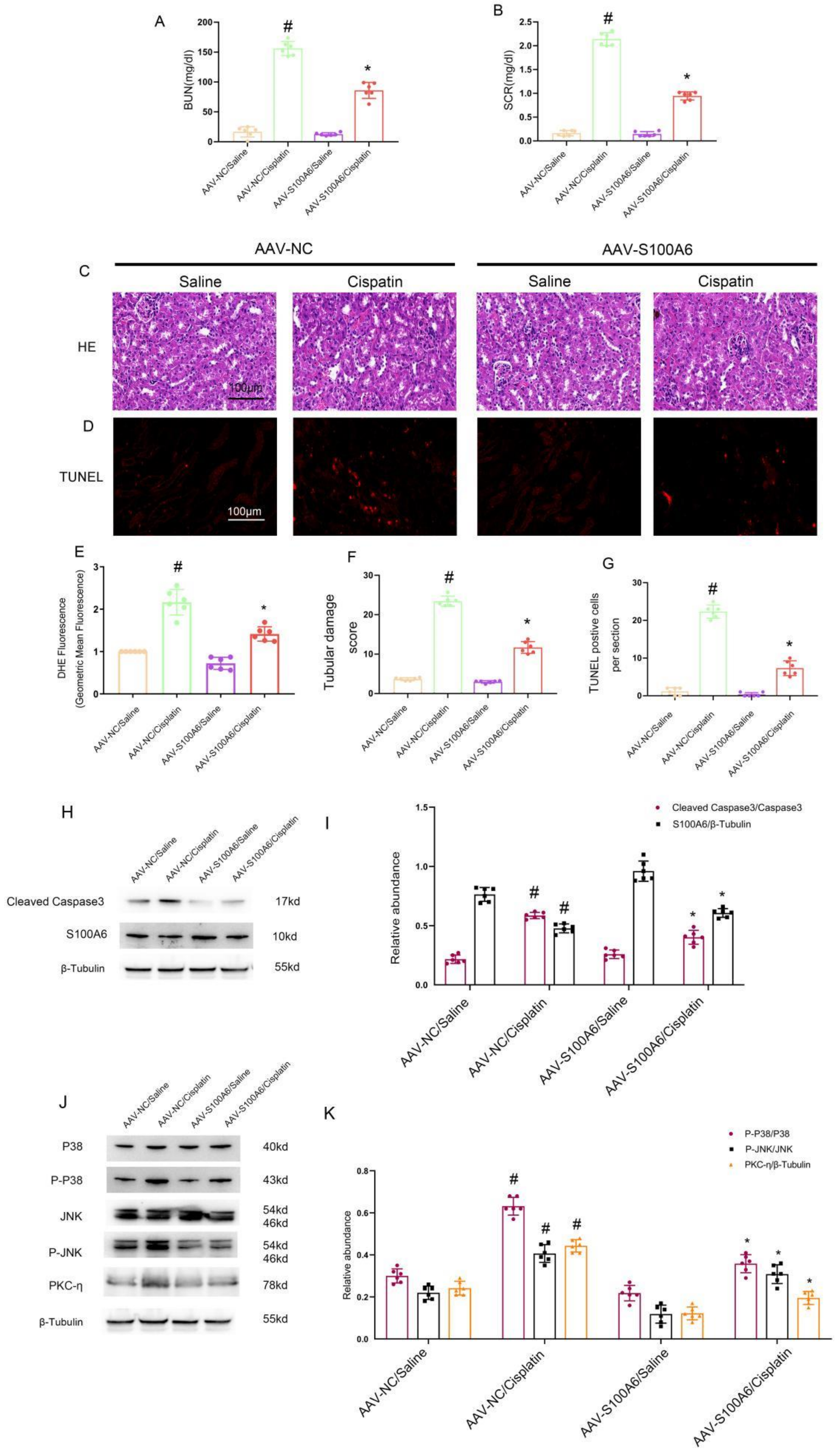
A
BUN(mg/dl)
B
SCR(mg/dl)
AAV-NC/Saline
AAV-NC/Cisplatin
AAV-S100A6/Saline
AAV-S100A6/Cisplatin
AAV-NC
AAV-S100A6
C
Saline
Cispatin
HE
100μm
D
TUNEL
100μm
E
DHE Fluorescence
(Geometric Mean Fluorescence)
F
Tubular damage
score
G
TUNEL postive cells
per section
H
Cleaved Caspase3
17kd
S100A6
10kd
β-Tubulin
55kd
I
Cleaved Caspase3/Caspase3
S100A6/β-Tubulin
Relative abundance
J
P38
40kd
P-P38
43kd
JNK
54kd
46kd
P-JNK
54kd
46kd
PKC-η
78kd
β-Tubulin
55kd
K
P-P38/P38
P-JNK/JNK
PKC-η/β-Tubulin
Relative abundance

Figure S12:S100A6 plasmids ameliorate cisplatin-induced renal injury and tubular cell apoptosis via inactivation of the PKC-η/ ROS/p38MAPK and JNK axes.

The C57BL/6 mice were injected with adeno-associated virus 2 (AAV2) carrying of S100A6 via renal artery once, after 3 days, and then injected intraperitoneally with cisplatin at 30 mg/kg, 0.9% saline was used as a control. (A) BUN. (B) Serum creatinine. (C) Hematoxylin and eosin staining. (D) Representative sections of TUNEL-positive cells. (E) Quantitative data for DHE (dihydroethidium). (F) Tubular damage score. (G) The number of TUNEL-positive cells. (H)Immunoblot analysis of cleaved caspase-3, PRDM16, S100A6, and β-tubulin. (I) Densitometry analysis of immunoblot bands. (J) Immunoblot analysis of P-P38MAPK, P38MAPK, P-JNK, JNK, PKC-η, and β-tubulin. (K) Densitometry analysis of immunoblot bands. Original magnification x 400. Scale bar: 100 µM. Data are expressed as the means ± SDs (n=6). # $P<0.05$ versus sham group. * $P<0.05$ versus cisplatin group.

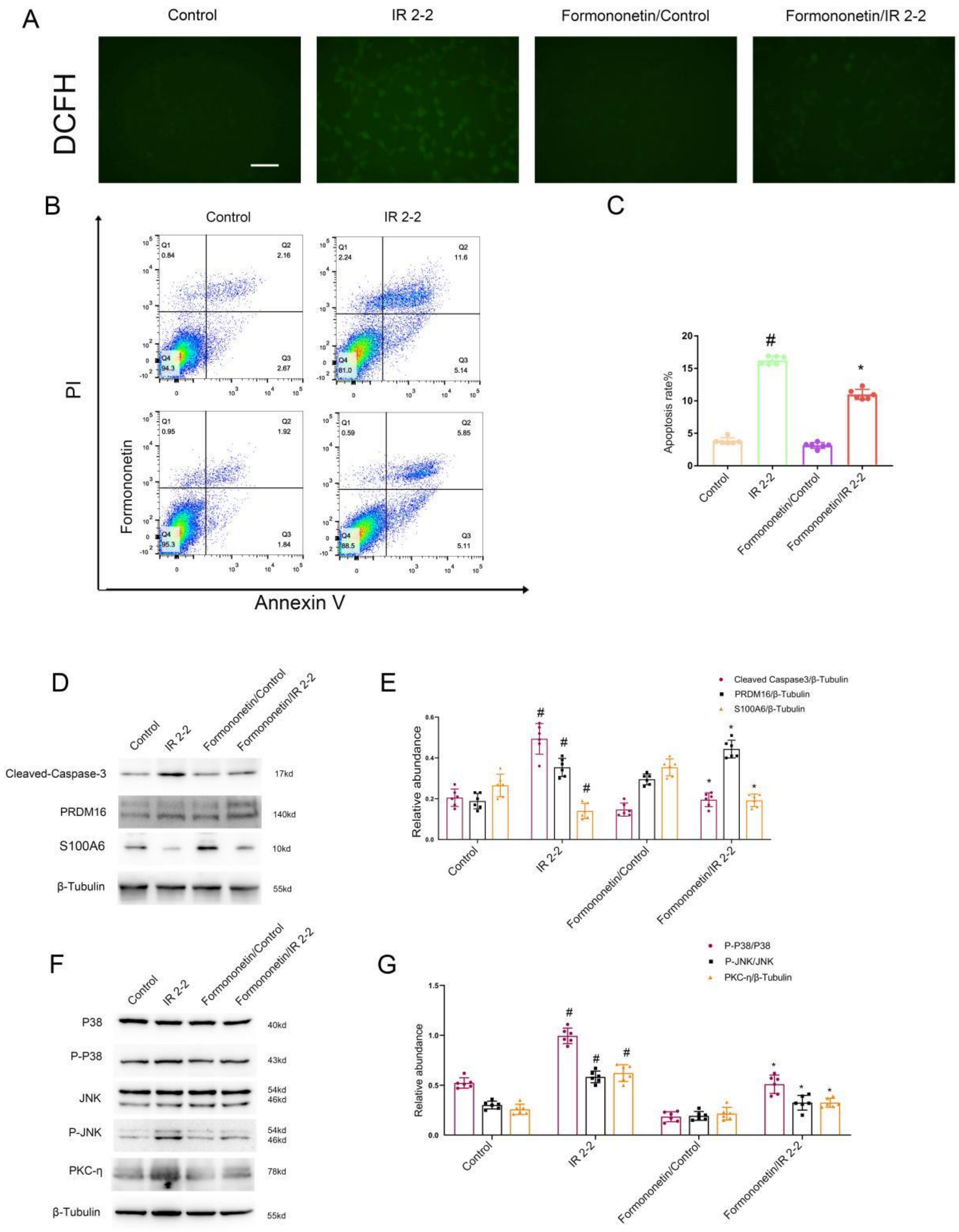

A
Control
IR 2-2
Formononetin/Control
Formononetin/IR 2-2
DCFH
B
Control
IR 2-2
PI
Formononetin
Annexin V
Q1 0.84
Q2 2.16
Q4 94.3
Q3 2.67
Q1 2.24
Q2 11.6
Q4 81.0
Q3 5.14
Q1 0.95
Q2 1.92
Q4 95.3
Q3 1.84
Q1 0.59
Q2 5.85
Q4 88.5
Q3 5.11
C
Apoptosis rate%
Control
IR 2-2
Formononetin/Control
Formononetin/IR 2-2
D
Control
IR 2-2
Formononetin/Control
Formononetin/IR 2-2
Cleaved-Caspase-3 17kd
PRDM16 140kd
S100A6 10kd
β-Tubulin 55kd
E
Cleaved Caspase3/β-Tubulin
PRDM16/β-Tubulin
S100A6/β-Tubulin
Relative abundance
Control
IR 2-2
Formononetin/Control
Formononetin/IR 2-2
F
Control
IR 2-2
Formononetin/Control
Formononetin/IR 2-2
P38 40kd
P-P38 43kd
JNK 54kd 46kd
P-JNK 54kd 46kd
PKC-η 78kd
β-Tubulin 55kd
G
P-P38/P38
P-JNK/JNK
PKC-η/β-Tubulin
Relative abundance
Control
IR 2-2
Formononetin/Control
Formononetin/IR 2-2

Figure S13: Formononetin ameliorated I/R-induced apoptosis in BUMPT cells via upregulation of PRDM16/S100A6 and inactivation of PKC-η/ **ROS/p38MAPK and JNK signal pathway**

The formononetin (20uM) were transfected into BUMPT cells and then exposed to ATP depletion for 2 hours and recovery for 2 hours. **(A)** ROS (reactive oxygen species) level assessed by DCFH(dichlorodihydrofluorescein). (B) Flow cytometry analysis. (C) quantitative data for apoptosis. (D)The immunoblot analysis of Cleaved Caspase3, PRDM16, S100A6, and β-tubulin. (E) Densitometry analysis of immunoblot bands. (F)The immunoblot analysis of P-P38MAPK, P38MAPK, p-JNK, JNK, PKC-η, and β-tubulin. (G) Densitometry analysis of immunoblot bands. Data are expressed as mean ± SD (n = 6). # $P<0.05$ versus scramble with Control group. * $P<0.05$ versus scramble with IR group.

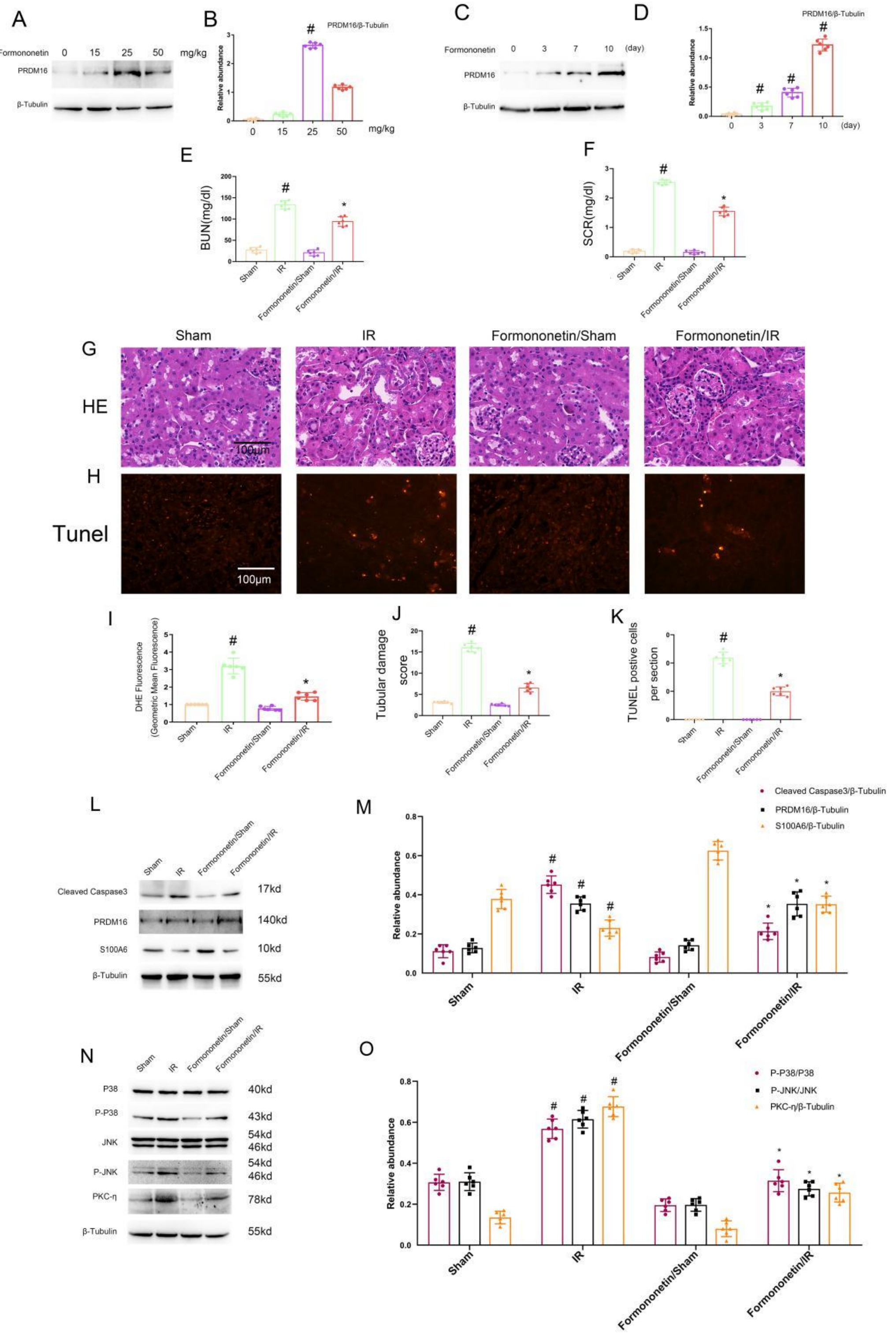
A
Formononetin 0 15 25 50 mg/kg
PRDM16
β-Tubulin
B
PRDM16/β-Tubulin
Relative abundance
0 15 25 50 mg/kg
C
Formononetin 0 3 7 10 (day)
PRDM16
β-Tubulin
D
PRDM16/β-Tubulin
Relative abundance
0 3 7 10 (day)
E
BUN(mg/dl)
Sham
IR
Formononetin/Sham
Formononetin/IR
F
SCR(mg/dl)
Sham
IR
Formononetin/Sham
Formononetin/IR
G
Sham
IR
Formononetin/Sham
Formononetin/IR
HE
100μm
H
Tunel
100μm
I
DHE Fluorescence (Geometric Mean Fluorescence)
J
Tubular damage score
K
TUNEL postive cells per section
L
Sham
IR
Formononetin/Sham
Formononetin/IR
Cleaved Caspase3 17kd
PRDM16 140kd
S100A6 10kd
β-Tubulin 55kd
M
Cleaved Caspase3/β-Tubulin
PRDM16/β-Tubulin
S100A6/β-Tubulin
Relative abundance
Sham
IR
Formononetin/Sham
Formononetin/IR
N
Sham
IR
Formononetin/Sham
Formononetin/IR
P38 40kd
P-P38 43kd
JNK 54kd 46kd
P-JNK 54kd 46kd
PKC-η 78kd
β-Tubulin 55kd
O
P-P38/P38
P-JNK/JNK
PKC-η/β-Tubulin
Relative abundance
Sham
IR
Formononetin/Sham
Formononetin/IR

Figure S14: Formononetin attenuated the IR-induced renal injury and renal cell apoptosis via regulation of PRDM16/S100A6/PKC-η/ ROS/p38MAPK and JNK axis

C57BL/6J male mice (8-10 weeks of age) were injected with formononetin at 0, 15, 25, 50mg/kg body weight for dose selection. and then were injected with formononetin at 25 mg/kg for 0, 3, 7, and 10 days. C57BL/6J male mice were clamped for 28min and then released for 48h to establish an IR model while treated with formononetin at 25 mg/kg. (A) The immunoblots analysis of the expression of PRDM16 and β-tubulin. (B) Densitometry analysis of immunoblot bands. (C) The immunoblots analysis of the expression of PRDM16 and β-tubulin. (D) Densitometry analysis of immunoblot bands. (E) BUN. (F) Serum creatinine. (G) Hematoxylin and eosin staining. (H) Representative sections of TUNEL-positive cells. (I) Quantitative data for DHE(dihydroethidium). (J) Tubular damage score. (K) The number of TUNEL-positive cells. (L)The immunoblot analysis of Cleaved Caspase3, PRDM16, S100A6, and β-tubulin. (M) Densitometry analysis of immunoblot bands. (N)The immunoblot analysis of P-P38MAPK, P38MAPK, P-JNK, JNK, PKC-η, and β-tubulin. (O) Densitometry analysis of immunoblot bands. Original magnification x 400. Scale Bar:100μM. Data are expressed as means ± SD (n=6). # $P<0.05$ versus sham group. * $P<0.05$ versus saline with IR group.

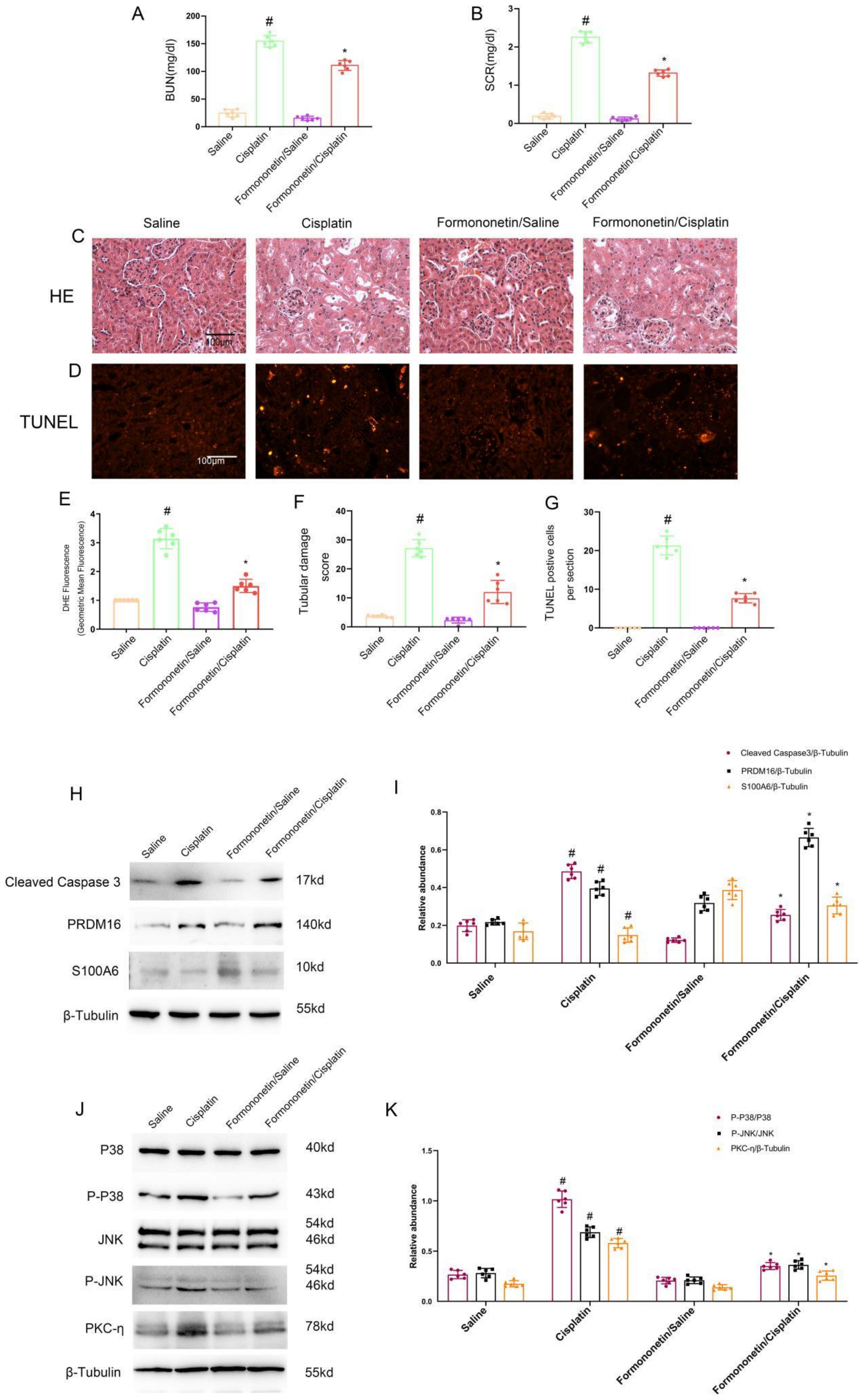

A
BUN(mg/dl)
Saline
Cisplatin
Formononetin/Saline
Formononetin/Cisplatin
B
SCR(mg/dl)
C
HE
D
TUNEL
100μm
E
DHE Fluorescence
(Geometric Mean Fluorescence)
F
Tubular damage score
G
TUNEL postive cells per section
H
Cleaved Caspase 3
17kd
PRDM16
140kd
S100A6
10kd
β-Tubulin
55kd
I
Cleaved Caspase3/β-Tubulin
PRDM16/β-Tubulin
S100A6/β-Tubulin
Relative abundance
J
P38
40kd
P-P38
43kd
JNK
54kd
46kd
P-JNK
PKC-η
78kd
K
P-P38/P38
P-JNK/JNK
PKC-η/β-Tubulin

Figure S15: Formononetin ameliorated the Cisplatin-induced renal injury and tubular cell apoptosis via regulation of PRDM16/S100A6/ PKC-η/ ROS/p38MAPK and JNK axis.

C57BL/6 male mice (8-10 weeks of age) were injected with 30 mg/kg body weight cisplatin and then with formononetin at 25mg/kg body weight, and saline ads a control. (A) BUN. (B) Serum creatinine. (C) Hematoxylin and eosin staining. (D) Representative sections of TUNEL-positive cells. (E) Quantitative data for DHE(dihydroethidium). (F) Tubular damage score. (G) The number of TUNEL-positive cells. (H)The immunoblot analysis of Cleaved Caspase3, PRDM16, S100A6, and β-tubulin. (I) Densitometry analysis of immunoblot bands. (J)The immunoblot analysis of P-P38MAPK, P38MAPK, P-JNK, JNK, PKC-η, and β-tubulin. (K) Densitometry analysis of immunoblot bands. Original magnification x 400. Scale Bar:100μM. Data are expressed as means ± SD (n=6). # $P<0.05$ versus saline group. * $P<0.05$ versus Cisplatin group.

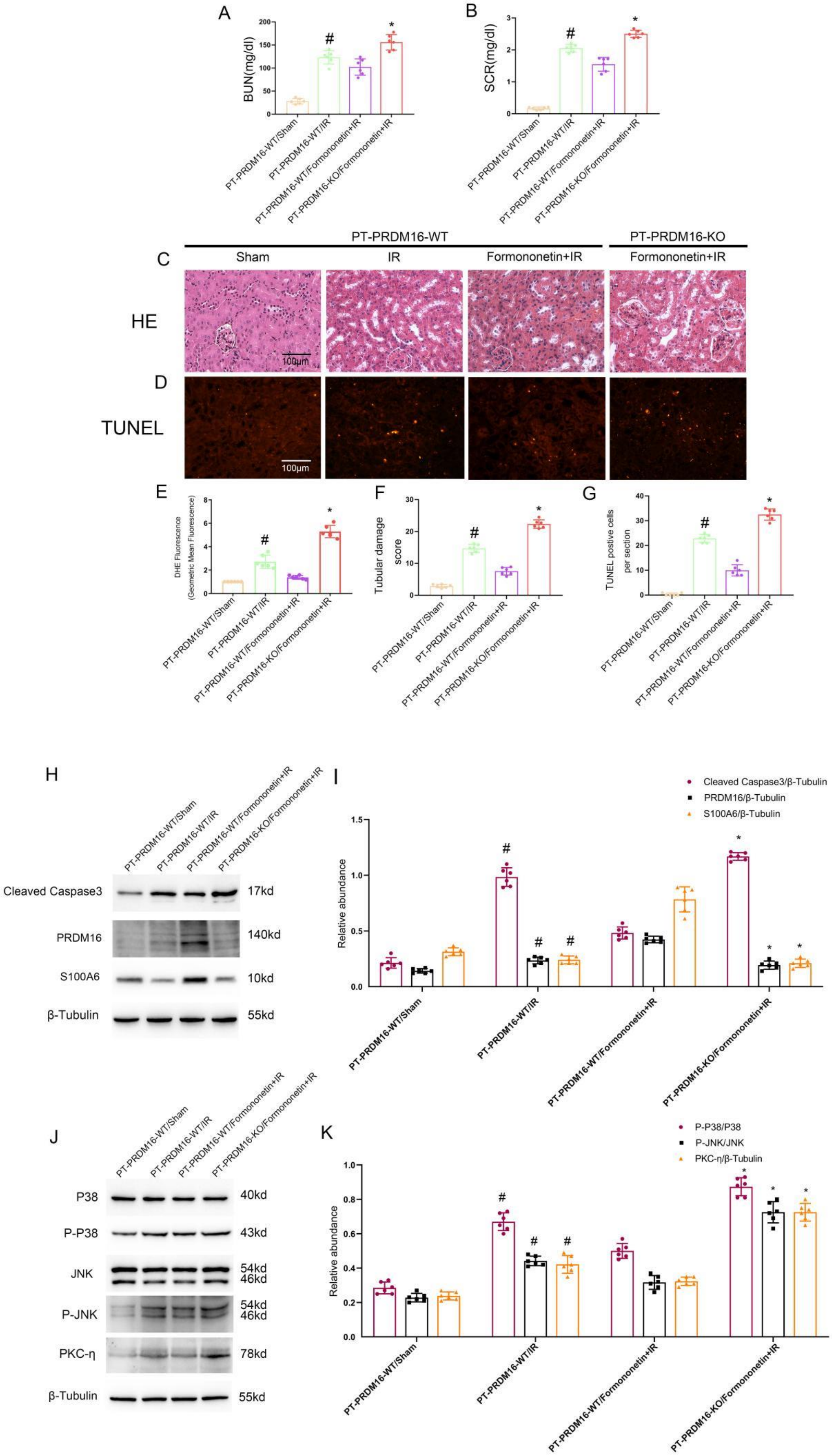

A
BUN(mg/dl)
B
SCR(mg/dl)
PT-PRDM16-WT/Sham
PT-PRDM16-WT/IR
PT-PRDM16-WT/Formononetin+IR
PT-PRDM16-KO/Formononetin+IR
PT-PRDM16-WT
PT-PRDM16-KO
Sham
IR
Formononetin+IR
C
HE
D
TUNEL
100μm
E
DHE Fluorescence (Geometric Mean Fluorescence)
F
Tubular damage score
G
TUNEL postive cells per section
H
Cleaved Caspase3
PRDM16
S100A6
β-Tubulin
17kd
140kd
10kd
55kd
I
Cleaved Caspase3/β-Tubulin
PRDM16/β-Tubulin
S100A6/β-Tubulin
Relative abundance
J
P38
P-P38
JNK
P-JNK
PKC-η
40kd
43kd
54kd
46kd
78kd
K
P-P38/P38
P-JNK/JNK
PKC-η/β-Tubulin

Figure S16:PT-PRDM16-KO enhanced the IR-induced renal injury and tubular cell apoptosis was not by Formononetin treatment.

The bilateral renal arteries of PT-PRDM16-KO and PT-PRDM16-WT littermate mice were clamped for 28 min and then released for 48h to establish an IR model while injected with formononetin at 25mg/kg body weight. (A) BUN. (B) Serum creatinine. (C) Hematoxylin and eosin staining. (D) Representative sections of TUNEL-positive cells. (E) Quantitative data for DHE(dihydroethidium). (F) Tubular damage score. (G) The number of TUNEL-positive cells. (H)The immunoblot analysis of Cleaved Caspase3, PRDM16, S100A6, and β-tubulin. (I) Densitometry analysis of immunoblot bands. (J)The immunoblot analysis of P-P38MAPK, P38MAPK, P-JNK, JNK, PKC-η, and β-tubulin. (K) Densitometry analysis of immunoblot bands. Original magnification x 400. Scale Bar:100μM. Data are expressed as means ±SD (n=6). # $P<0.05$ versus sham group. * $P<0.05$ versus IR group.

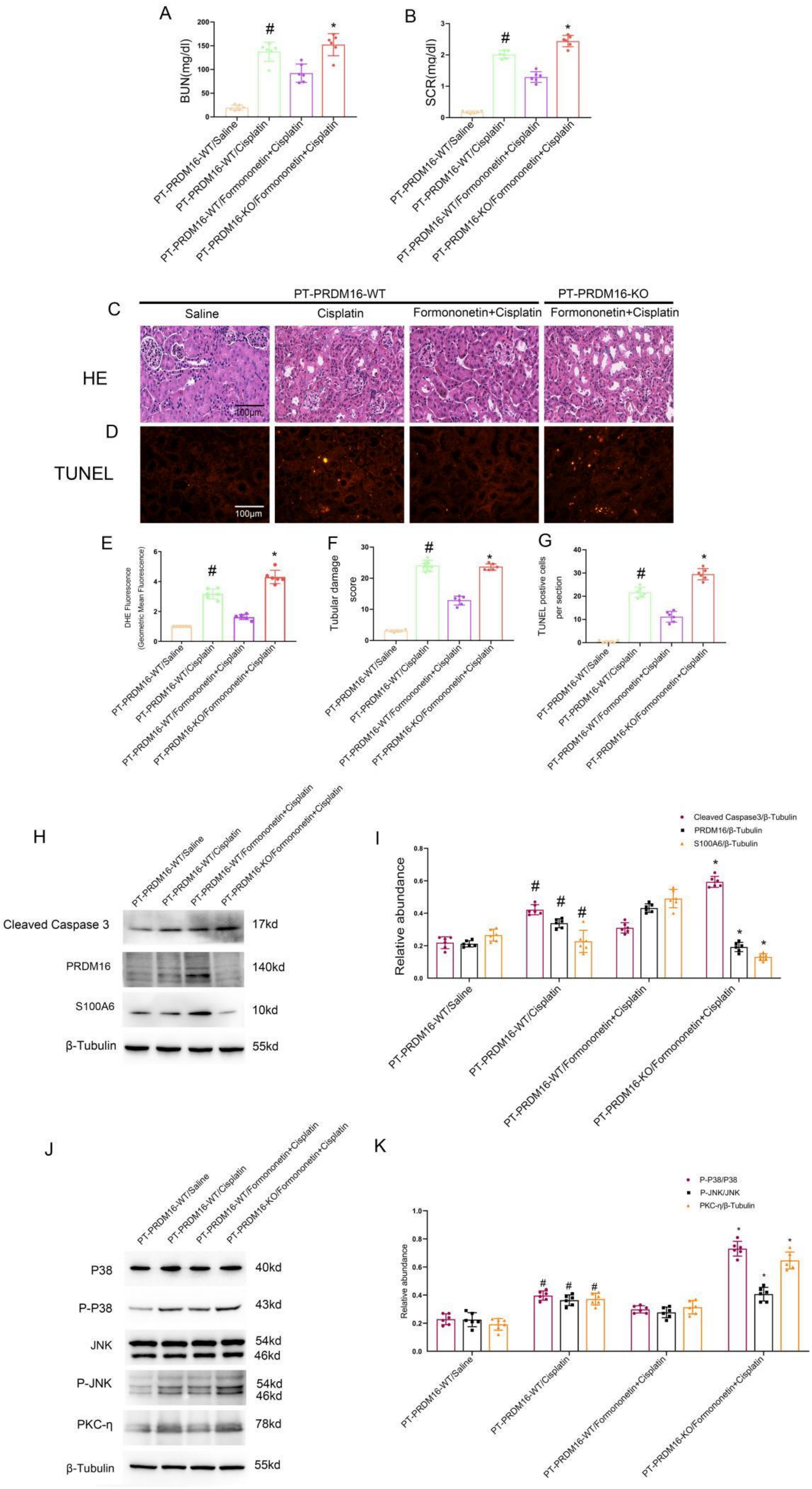
A
BUN(mg/dl)
PT-PRDM16-WT/Saline
PT-PRDM16-WT/Cisplatin
PT-PRDM16-WT/Formononetin+Cisplatin
PT-PRDM16-KO/Formononetin+Cisplatin
B
SCR(mg/dl)
C
PT-PRDM16-WT
PT-PRDM16-KO
Saline
Cisplatin
Formononetin+Cisplatin
HE
100μm
D
TUNEL
E
DHE Fluorescence (Geometric Mean Fluorescence)
F
Tubular damage score
G
TUNEL postive cells per section
H
Cleaved Caspase 3
PRDM16
S100A6
β-Tubulin
17kd
140kd
10kd
55kd
I
Cleaved Caspase3/β-Tubulin
PRDM16/β-Tubulin
S100A6/β-Tubulin
Relative abundance
J
P38
P-P38
JNK
P-JNK
PKC-η
40kd
43kd
54kd
46kd
78kd
K
P-P38/P38
P-JNK/JNK
PKC-η/β-Tubulin

Figure S17:PT-PRDM16-KO enhanced the Cisplatin-induced renal injury, tubular cell apoptosis was not by Formononetin treatment.

The bilateral renal arteries of PT-PRDM16-KO and PT-PRDM16-WT littermate mice were injected intraperitoneally with cisplatin at 30 mg/kg while injected with formononetin at 25mg/kg body weight. (A) BUN. (B) Serum creatinine. (C) Hematoxylin and eosin staining. (D) Representative sections of TUNEL-positive cells. (E) Quantitative data for DHE(dihydroethidium). (F) Tubular damage score. (G) The number of TUNEL-positive cells. (H)The immunoblot analysis of Cleaved Caspase3, PRDM16, S100A6, and β-tubulin. (I) Densitometry analysis of immunoblot bands. (J)The immunoblot analysis of P-P38MAPK, P38MAPK, P-JNK, JNK, PKC-η, and β-tubulin. (K) Densitometry analysis of immunoblot bands. # $P<0.05$ versus Saline group. * $P<0.05$ versus Cisplatin group. ∧$P>0.05$ versus Cisplatin group.

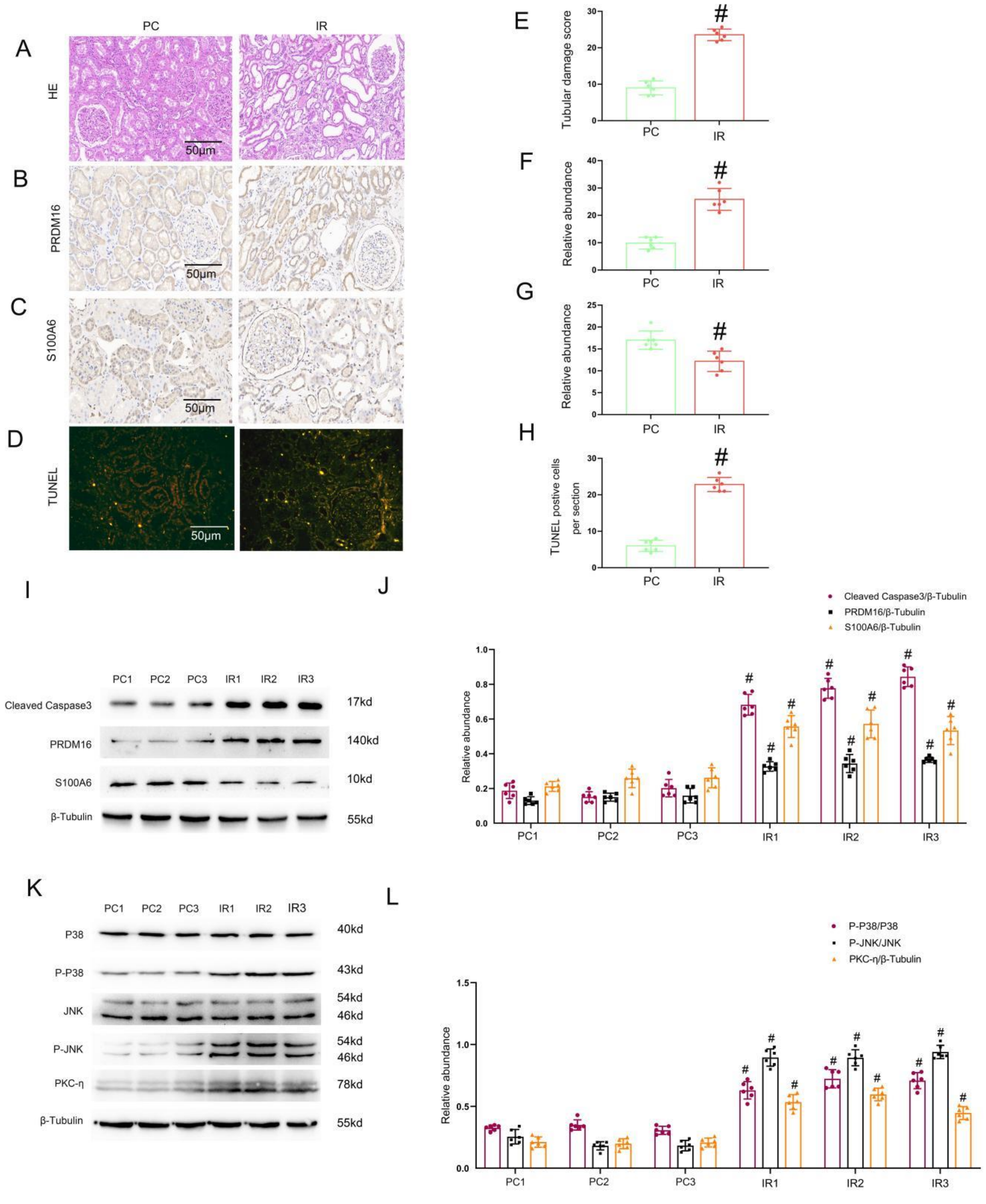

Figure S18: PRDM16/S100A6 axis are involved in the progression of patients's AKI.

(A) Hematoxylin and eosin staining. (B) immunohistochemical staining of PRDM16. (C) immunohistochemical staining of S100A6. (D) Representative sections of TUNEL-positive cells. (E) Tubular damage score. (F) Quantification analysis of PRDM16

staining (G) Quantification analysis of S100A6 staining (H)The number of TUNEL-positive cells. (I)The immunoblot analysis of Cleaved Caspase3, PRDM16, S100A6, and β-tubulin. (J) Densitometry analysis of immunoblot bands. (K)The immunoblot analysis of P-P38MAPK, P38MAPK, P-JNK, JNK, PKC-η, and β-tubulin. (L) Densitometry analysis of immunoblot bands. Original magnification x 400. Scale Bar:100μM. Data are expressed as means ± SD (n=6). # *P*<0.05 versus PC group.

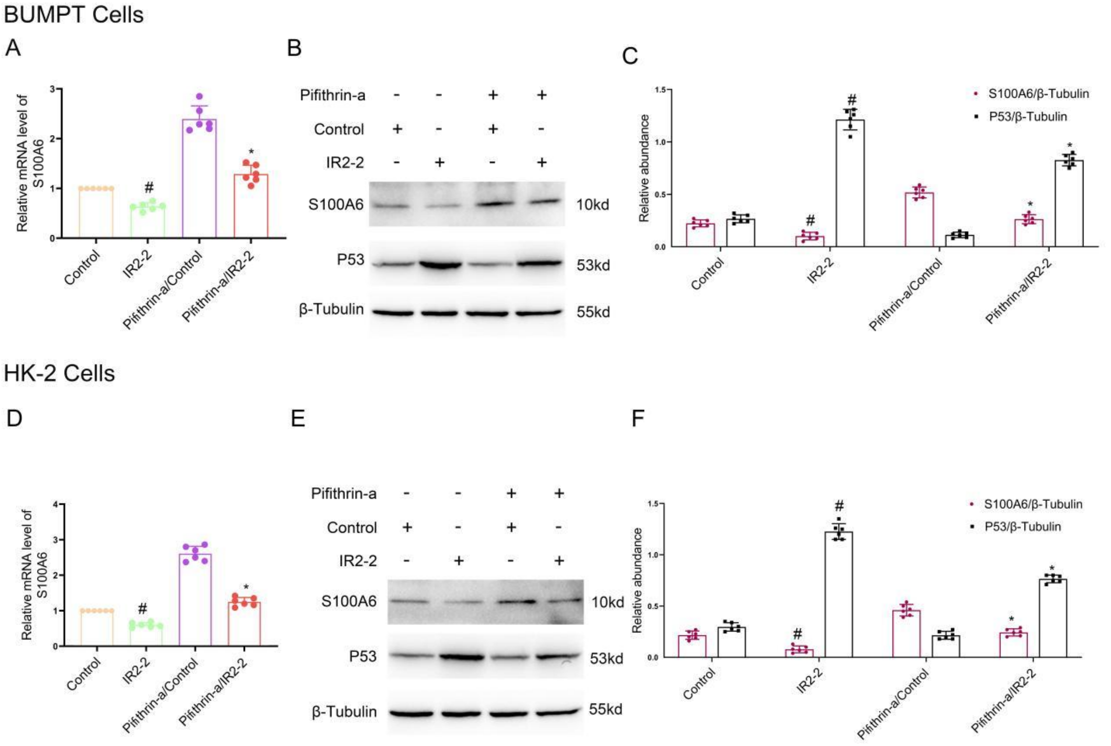

Figure S19:Pifithrin-a enhanced the expression of S100A6.

The formononetin (20uM) were transfected into BUMPT cells or HK-2 cells and then exposed to ATP depletion for 2 hours and recovery for 2 hours. (A) RT–qPCR analysis of S100A6. (B)Immunoblot analysis of S100A6, P53, and β-tubulin. (C) Densitometry analysis of immunoblot bands. (A) RT–qPCR analysis of S100A6. (B)Immunoblot analysis of S100A6, P53, and β-tubulin. (C) Densitometry analysis of immunoblot bands. Data are expressed as means ± SD (n=6). # *P<0.05* versus scramble with Control group. * *P<0.05* versus scramble with IR group.

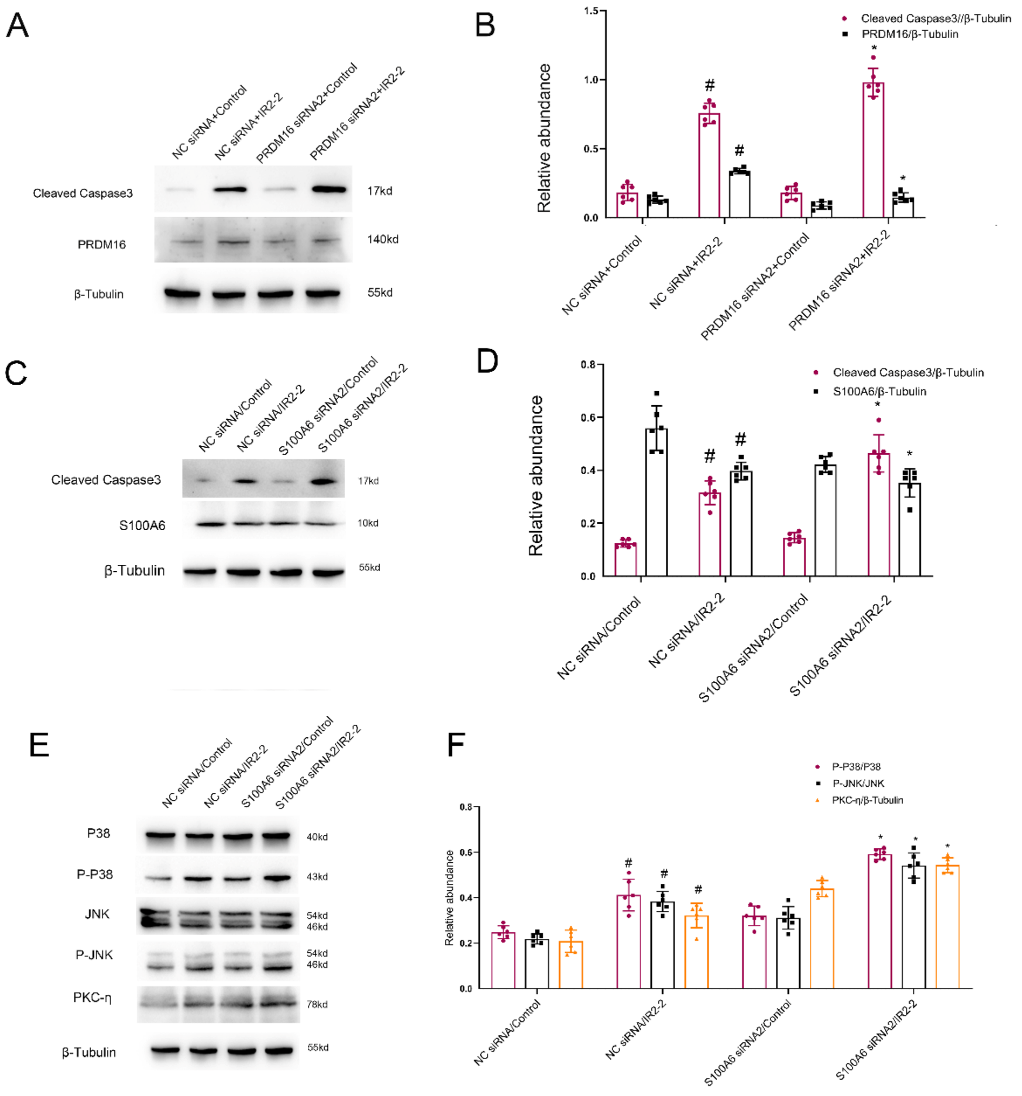

Figure S20: PRDM16 and S100A6 negatively regulates the I/R-induced expression levels of apoptosis in BUMPT cells.

The PRDM16 siRNA2 or The S100A6 siRNA2 were transfected into BUMPT cells, which were then exposed to ischemia for 2 hours and recovery for 2 hours. (A)Immunoblot analysis of cleaved caspase3, PRDM16, and β-tubulin. (B) Densitometry analysis of immunoblot bands. (C)Immunoblot analysis of cleaved caspase3, S100A6, and β-tubulin. (D) Densitometry analysis of immunoblot bands. (E) Immunoblot analysis of P-P38MAPK, P38MAPK, P-JNK, JNK, PKC-η, and β-tubulin. (F) Densitometry analysis of immunoblot bands. Data are expressed as means ± SD (n=6). # *P<0.05* versus scramble with Control group. * *P<0.05* versus scramble with

IR group.

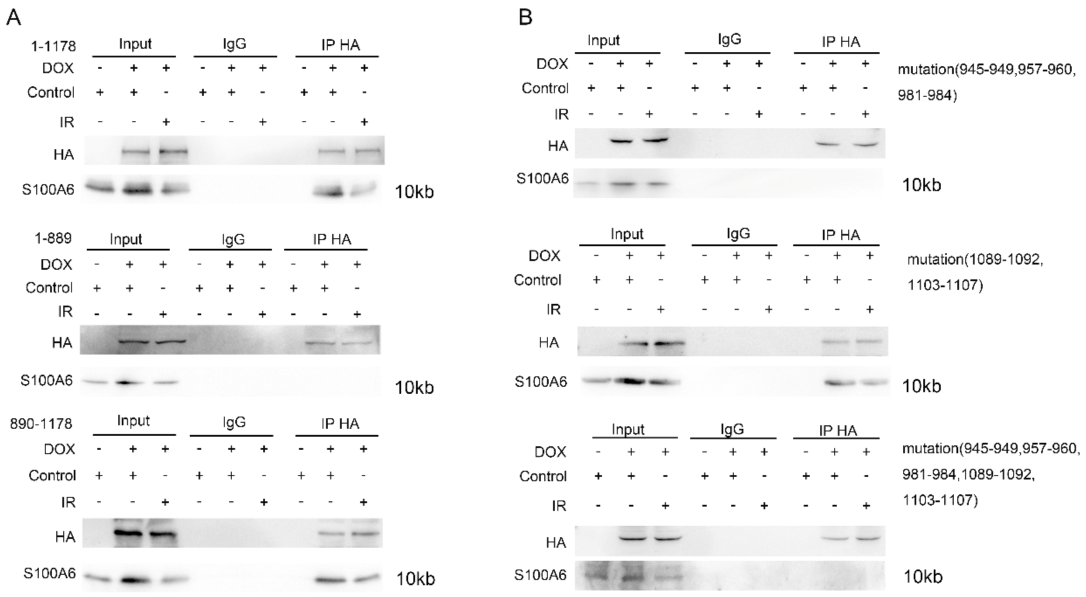

Figure S21:PRDM16 positively regulates and interacts with S100A6 in BUMPT cells under baseline and I/R treatment conditions

(A-B) Anti-HA immunoprecipitates were analyzed for HA and then detected for S100A6 using immunoblotting.

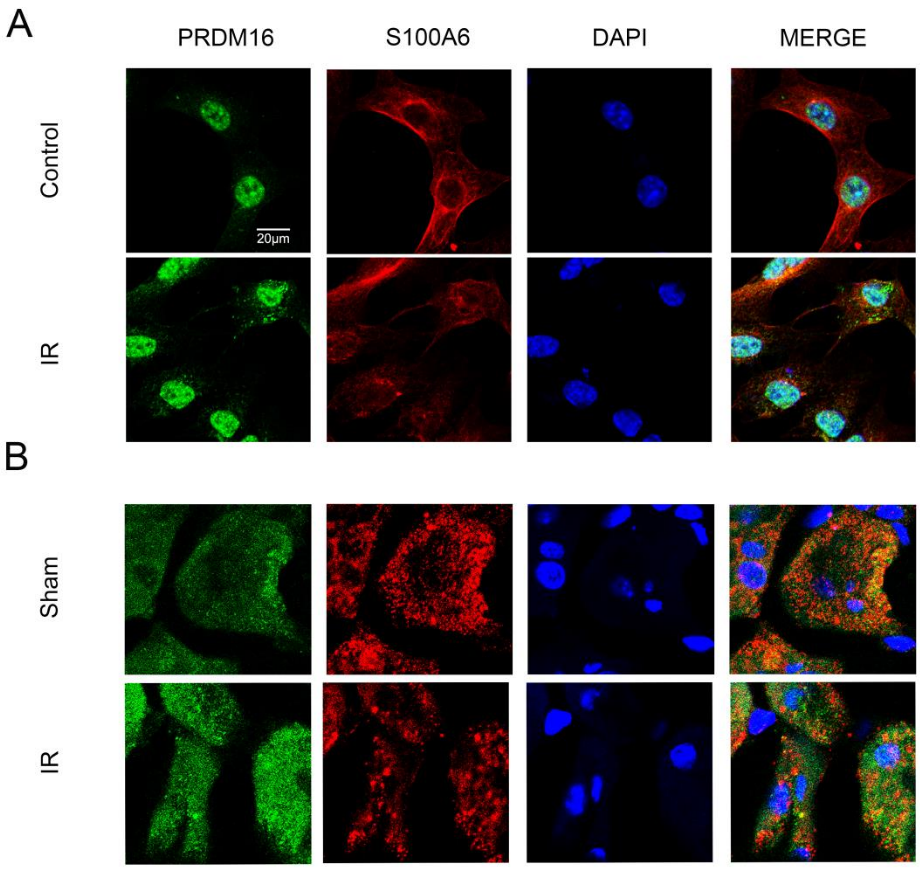

Figure S22:Colocalization of PRDM16 and S100A6 in BUMPT cells and kidneys of AKI mice.

(A) Localization of PRDM16 with S100A6 in BUMPT cells with or without ischemia (2 h) and recovery (2 h). (B) Localization of PRDM16 with S100A6 in BUMPT cells in the kidneys of the mouse model in the sham and I/R groups. Original magnification x600. Scale Bar:20μM.

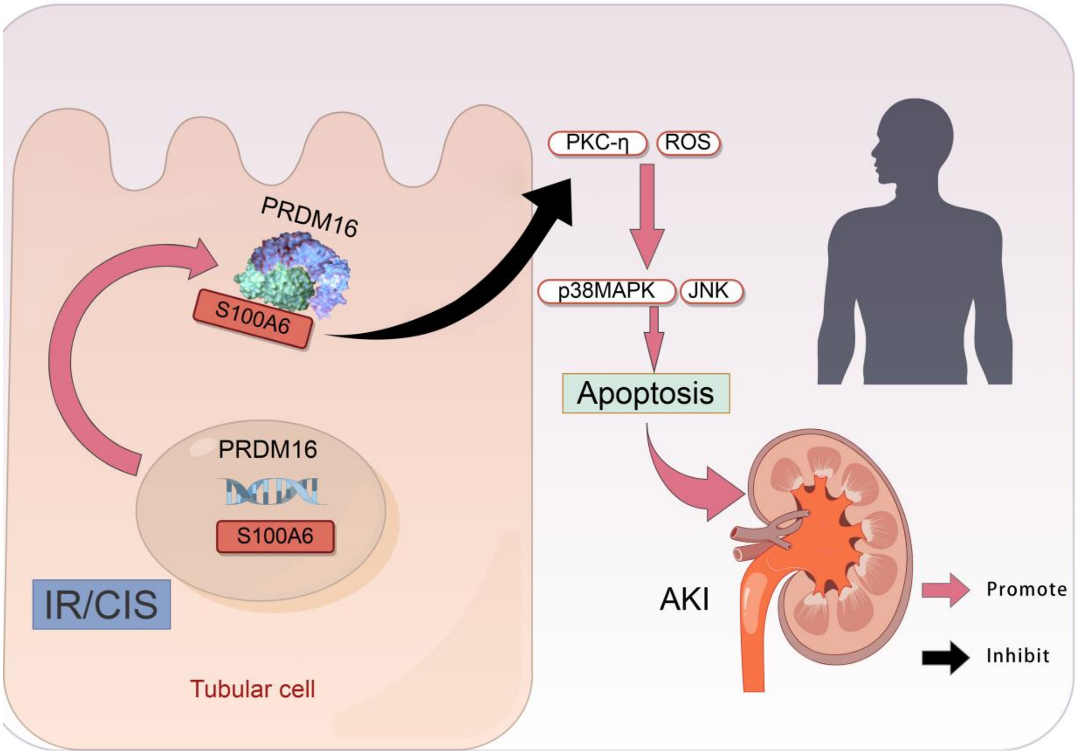

Figure S23 : The anti-apoptosis pathway of PRDM16/S100A6/PKC-η/ROS/p38MAPK and JNK axes activated in BUMPT cells and HK-2 cells.

Table S1 Upregulation proteins of PRDM16

HA-Vector

Blmh	Hspb1	Tert	Odf2
Gm5478	2210010C04Rik	Eef1b	DSP

HA-PRDM16-Plasmid

Rab11b	Hnrnpab	Tert	Gm5478
9130019P16Rik	Rps25	Odf2	Npm1
Dctn2	Hsp90aa1	Nup85	2210010C04Rik
Myof	Rpl5	Sfn	Krt76
Atp5c1	Blmh	Tubb2b	Eef1b
P4hb	Hspb1	Ccdc187	DSP

Atp1a1	Tagln2	Adam19	
4930544G11Rik	Arg1	S100a6	

Table S2 **S100A6 promoter**

-2000--1500

AAATAATAATCTGTAATTCATACCAATTCCTACTGAAAACAGCTTGAGGTCT
GGCTATGTGGCCCTGAGCATCATCCTGAGCTAAGTCCTAGACTTATCCTGCT
TGCACTGTAAGGCCAAGCTGGTAAAGGATCCAGACATCACCACTCTCTGTA
AGATCAGTATACTGGAACTGAGAACCCAGCATGCCCCCTACCCTAAAACCT
GGCCTGGCACCAGATGTCCTCCTCCCAGTTTGGTCCCTGCACCCTGGCTCT
ACTAGGAAGTTGAAGACAGAGCCTTAGGGTAAA
ACAAGGAGATGCAAATTTGTAGACCCTGCTTTCCCTCCATTGCAATCAGTTC
TAGGTCTCCCTTGGATGCAGGATGTTGCTAGTCATGATGCTGGAAACTGCTT
CTCTGGAACACGATCCTGGTCCCCAGACTGGGCTCCTCCCCTGGGGCTCTG
TCCCCCTAATTAGCACCAGATGAACCAGAGGTCCTCCCCTCTACCTTTATTA
GCT

-1500--1000

AGACCACCTGTGTGTGGCCCAAGGCTTAGTGGTCCAAAATGTCTGTTCTCC
CCTGCCTCAGCCAAGACACAAACCACCTTAAAACCAGTGCGTGTAATGGAT
AAGGCTTTATTTCTCTGCTGGAAGGATCCATCTGGGAGAAAGAGAAGGGC
AGGGGCCATAGGAGGGGCAGTTAAAGAGGCTGCAGAGGTCAGGATCAGG
AGCTGCCAGTGTCCTGGTCATTTGTTGTCCTCTAGGAAGAAGTCATTGTAG
GCCATGCACAGCGTGGTCAGGAACACAGAGTACTCCTTGAAGTCAATCTCC
TGGTCGCTGTTTTTGTCCAGGCTCTTCATCAAGTTATCAATGCTGCTCTCCT
TCATCTTCTACAAGGGCCCAAGAGGAGGAGACTACTCAGCAGCCCAGCCC
CAGCCCACACCTTTACCACAGGACCATATTCCAAATGAATTACAGTAGGAA
GGCTTAGTCTAGCCTTTGCTCCTGGGACATCTTC
CCATCCAAAG

-1000--500

CACTCACTGCCTGGATTTTCATCCTTAGAGCAGCTTATGCATGAATGTGCAC
ATAGTAGAGCCAAGCCCAGCGAGCATTCTCAGTTACTCATCTCAAGTGAAA
AGTTTTGAACATTTTCTCCTGGGGTGGGGGGGGGTAATGGGGAGGAGTCTT
GAGAAGAGGAAGTTGGTAGGGTGTGGGGAGGGATTAGCATCCTGAAGTCA
CTCTTCAGTGAGTAATGGGAGGGGTGTTCCTGGAAGGGAGAGGAGAAGGG
CTCCTGGGGTGGTCTCCTGCGGGCTATCCCCAAGTC
AGACCTGCGGGACCATCACTGCTGGGCCACAGCCCATCCCTCTGGCCTCCT
AGCTCTCACGGCCTTTTCTCTTGTTTTCTCTGCATCTTTGCTGTCTGGGCAG
CTTTGTTATTTTACAGGAGTGCAGAACAAAGTCAGGACACCTCCAACAATG

TCCATGATGAAGGACTGCCAGTGAAGAGGGATGAGAGCCCAGCTCCTGCT
ATCCAC

-500-0

CAGCCCGAGAGCAGTGTCCTTAAAGTTAGCATCAGTGTGAGGGGGACTGG
GAACCACACTGATGCTGTTGCCCTCTAGTGGCAAGGTCCCCAAGTGAGGG
AGGGGAAGCAGTCTGTCTACCACATCCTCTAAGAAATGGTTCTCACTCAAC
CTGACTAATGCTGCGGCCCTTTAATACTGTTCTTCAAGTTGTGGTGGTCCCC
CAATCATAAAGTTATTCTCGTTTCTACTTCATAGCTATAATTTTGCTACTGTTA
TGAATCTTAATGTGTATATCTGTGTTTTCAGATGGTCTTAGGCAATTCCTGTG
AAAGGGGTCTCAACCCACAGGTTGAGAACCACTGGTCTAAATCTTGATATA
TAGCTAAAGACGTTGATCCCAAAAGGTAGAATTTTGAGCCTTGTATCAAAG
GGGGCATGGGGAAAGAACACAGTCTAAAAGATTTTAAACAGCATCCCCCA
CCAACCATAAATTTCTAGCAACATCACTCGCCTCCTCC
